# Using next-generation sequencing to unravel the diet composition of Burrowing Parrots over the annual cycle

**DOI:** 10.1007/s00114-026-02159-3

**Published:** 2026-09-24

**Authors:** Juan F. Masello, Mauricio Failla, Cintia V. Leder, Petra Quillfeldt

**Affiliations:** 1 Faculty of Biology and Chemistry, Justus Liebig University Giessen, Giessen, Germany; 2 Department of Biological Sciences, University of Venda, Thohoyandou, Republic of South Africa; 3 Proyecto Patagonia Noreste, Balneario El Cóndor, Río Negro, Argentina; 4 Sede Atlántica, Centro de Estudios Ambientales desde la NorPatagonia (CEANPa), Universidad Nacional de Río Negro, Viedma, Argentina; 5 Consejo Nacional de Investigaciones Científicas y Técnicas de Argentina (CONICET), Buenos Aires, Argentina; 6 Department of Evolutionary Population Genetics, Bielefeld University, Konsequenz 45, Bielefeld, 33615 Germany

**Keywords:** Diet composition, Metabarcoding, Molecular scatology, Non-invasive, Non-metric multidimensional scaling, Parasite

## Abstract

Understanding the diet of an organism is crucial for disentangling its role in ecosystem function, and to unravel foraging behavior, population dynamics, competitive interactions among species and evolutionary processes. Furthermore, diet studies can be useful to better interpret environmental change and its impact on wildlife. Nevertheless, studying the diet of organisms that forage over large areas can be challenging. In such cases, next-generation sequencing of DNA in fecal samples can be used to identify dietary items consumed. Using this methodology, we investigated the diet composition of the Burrowing Parrots *Cyanoliseus patagonus* from the El Cóndor colony, north-eastern Patagonia, Argentina. Over the annual cycle, we collected fecal samples at the roosting place used by these birds every night. We identified a large variety of plant molecular operational taxonomic units (MOTUs) in the diet of Burrowing Parrots throughout the year. Remarkably, we found that only 13% of plant MOTUs consumed were native plants. This was unexpected, suggesting a shift from a predominantly native plant-based diet in the past to a diet that is now primarily composed of introduced plants. This shift could demonstrate the behavioral flexibility of this species in adapting to current severe environmental degradation in north-eastern Patagonia. Alternatively, it could be a sign of a suboptimal strategy that could negatively impact on the persistence of the El Cóndor colony, the world’s largest colony of Psittaciformes. Additionally, this methodology enabled the identification of parasites not previously recorded in the digestive tracts of Burrowing Parrots (Gongylonematidae, Hymenolepididae, Onchobothriidae, and Capillariidae).

**Supplementary Information:**

The online version contains supplementary material available at https://doi.org/10.1007/s00114-026-02159-3.

## Introduction

A detailed knowledge on the diet composition of an organism is vital to understand its role in ecosystem function (Barnagaud et al. 2019). It is also crucial to disentangle evolutionary processes, as diet is under strong selection due to its relationship with individual fitness, population dynamics, and the competitive interactions among related species (Grant and Grant 2006; Rivera et al. 2019; Masello et al. 2021). Diet composition data is also needed for a proper understanding of foraging behavior (Masello et al. 2021), as well as the occurrence and abundance of prey species (Sutton et al. 2021). Furthermore, this knowledge allows a better interpretation of environmental change and its impact on wildlife (Estes et al. 2011; Sydeman et al. 2015; Cabodevilla et al. 2021; Grames et al. 2023), thereby facilitating adequate population management (Brett and Ortiz-Catedral 2021; Oliveira et al. 2021).

The importance of understanding the dietary habits of specific species has prompted extensive scientific research. Nevertheless, the diet of some organisms remains difficult to investigate in detail. For instance, the diet of small, nocturnal, subterranean, underwater, oceanic, or elusive species can be challenging to investigate (Pompanon et al. 2012; Traugott et al. 2013). To circumvent this obstacle, researchers have employed various methodologies such as microscopic examinations of feces, high-resolution photography, lethal sampling, induced regurgitation, and stomach flushing (Deagle et al. 2007; Michalik et al. 2010; Gaglio et al. 2017). Some of these visual methodologies are invasive, others demand considerable labor, some lack resolution, and many are restricted to assessing diet over short time frames (Deagle et al. 2007; Pompanon et al. 2012; Gaglio et al. 2017). More recently, DNA-based methods, particularly next-generation sequencing (NGS), have provided non-invasive alternatives that allow for the identification of dietary items through the characterization of the DNA in gut or fecal samples (Swift et al. 2018; Alberdi et al. 2019; Deagle et al. 2019). Feces have become the most widely used sample type (Deagle et al. 2010, Dunn et al. 2018; Rytkönen et al. 2019; Masello et al. 2021, 2023). Furthermore, our understanding of the diets of many vertebrates is also limited because research is often only conducted during the breeding season (Marra et al. 2015). This is partly due to the logistical challenges of collecting annual data, especially when it is necessary to track animals over vast distances throughout the year (Marra et al. 2015). The use of NGS of DNA in fecal samples also provides an alternative method for investigating temporal variations in animal diets (Cabodevilla et al. 2021; Tang et al. 2023; Coomes et al. 2025).

The Burrowing Parrot (*Cyanoliseus patagonus*) is a Psittaciformes bird breeding in Argentina and Chile. Particularly in Argentina, these parrots live in the dry landscapes of the northern and southern ‘Monte’, the ‘Chaco Seco’, the Patagonian steppes, and the ‘Espinal’ ecoregions. However, they are always found close to water because they need to drink several times per day (Masello et al. 2011, 2015). Burrowing Parrots breed in colonies and dig burrows in soft sandstone, limestone or earthen cliffs, also known as “barrancas” (gorges or ravines) (Masello et al. 2006a; Ramirez-Herranz et al. 2017; Lera et al. 2023a; b). The species’ largest colony, which is also considered to be the largest Psittaciformes colony in the world, is located near to El Cóndor village, next to the Río Negro estuary in north-eastern Patagonia, Argentina (Masello et al. 2006a, 2025; Llanos et al. 2011). The colony extends 18.1 km and comprises 71% of the global breeding population of the species. Over a long-term monitoring of the colony (1998 to 2019), the total population size varied between 22,000 and 41,000 active nests (Zanellato et al. 2024; Masello et al. 2025). The El Cóndor colony has been subject to significant human-induced disturbance (detailed in Masello et al. 2025). The main current threats are the ongoing clearance of the ‘Monte’ vegetation (Pezzola et al. 2004; Masello et al. 2006a, 2025; Boeri et al. 2014; Cosentino et al. 2021) and the increased urbanization and related infrastructure in the vicinity of the colony (Masello et al. 2025).

Information gathered opportunistically, suggests that the diet of Burrowing Parrots in the northern ‘Monte’ (i.e. about 1000 km NW from El Cóndor colony) comprises seeds and fruits of native trees and grasses: Coast Sandbur *Cenchrus spinifex*, Chañar *Geoffroea decorticans*, Caldén *Prosopis caldenia* (= *Neltuma caldenia*), Chilean Algarrobo *P. chilensis* (*= N. chilensis*) and Sweet Algarrobo *P. flexuosa* (= *N. flexuosa)* (Bucher et al. 1987; de la Peña 2011). In the Patagonian Andes, Hudson (1923) reported berries of the native subshrub *Empetrum rubrum* and in other parts of Patagonian seeds of the introduced milk thistle *Silybum marianum* as part of the Burrowing Parrot diet. Wetmore (1926) added berries of the native shrubs *Lycium* sp. and *Discaria* sp., while de la Vega (2003) mentioned the berries of a third native shrub *Schinus johnstonii*. Previous studies near the El Cóndor colony reported that Burrowing Parrots regularly consumed the seeds of non-native plants, such as the Bathurst Burr *Xanthium spinosum*, the Musk Thistle *Carduus thoermeri*, the Wild Oat *Avena fatua*, and the Curled Dock *Rumex crispus.* The parrots also consumed berries from the native shrub Piquillín *Condalia microphylla* (Masello et al. 2006a; Sánchez et al. 2016). Forshaw (1989) mentioned Burrowing Parrot preference for soft plant parts. In line with this, we observed buds and other soft vegetable matter in the crop contents of Burrowing Parrot nestlings (Masello and Quillfeldt 2004). In some regions of Argentina, Burrowing Parrots are blamed as an agricultural pest. The crops that have been reported as affected include maize, barley, sunflowers, wheat, millet, almonds, apples, peaches, walnuts, and vineyards (Bucher and Bedano 1976; Bucher 1984; de la Peña 2011). However, systematic scientific evaluations have not been conducted until recently. In the El Cóndor region, some farmers frequently claim that maize is greatly affected, followed by sunflowers, oats, wheat and millet (Failla et al. 2008). A previous study, however, found that only 16% of farmers who were asked mentioned that the Burrowing Parrot actually damaged their crops (Failla et al. 2008). Furthermore, detailed research covering the foraging areas used daily by the Burrowing Parrot from El Cóndor (approximately 250,000 hectares; Masello et al. 2006a) revealed that damage to field crops was very limited (Sánchez et al. 2016). The parrots affected only 0.1 to 0.4% of the sunflower harvest, leaving wheat and maize undamaged (Sánchez et al. 2016). They primarily consumed grain that had been spilled on the ground during harvesting, as well as unharvested grain from cultivated pastures, which was mostly found on road margins (Sánchez et al. 2016). These previous results and anecdotal information suggest that the Monte vegetation plays a significant role in the diet of Burrowing Parrots, and that there is little interaction with the crops in the El Cóndor region. However, a detailed study of the species’ diet components was lacking until now.

In this study, we investigated the diet composition of the Burrowing Parrots from El Cóndor, north-eastern Patagonia, Argentina, using DNA metabarcoding of fecal samples collected over the entire annual cycle. With the detailed and accurate insights gained, we aim to improve our understanding of the species feeding ecology, which would facilitate the management and protection of the natural resources they need.

## Materials and methods

### Sample collection and study area

From January to December of 2021, we collected fresh fecal samples from Burrowing Parrots roosting in the village of El Cóndor, north-eastern Patagonia, Argentina. Each evening, large flocks of Burrowing Parrots roost mainly on the power lines inside the village, located at a distance of 1,000 to 2,200 m from the easternmost part of the El Cóndor Burrowing Parrot colony (Masello et al. 2006a, 2025). This is the only known roosting place, within a radius of 30 km, associated with the El Cóndor Burrowing Parrot colony (Masello et al. 2006a).

We collected 220 fresh fecal samples in total, corresponding to 20 samples per month, except for February. No parrots roosted in the village during February, and thus no samples were obtained. Each year, towards the last days of January, the Burrowing Parrots from the El Cóndor colony cease roosting in the village of El Cóndor, and are not seen in the surrounding natural areas. In the first days of March, they return and resume their habit to roost in the village of El Cóndor (authors’ pers. observ.). Their movements in February and their activities during that time are unknown.

We placed a large piece of clean metal foil below the power lines to collect the fresh scat samples. To avoid contamination, we took special care to sample the central part of the feces and avoid the part that was in direct contact with the metal sheet. The samples were immediately placed in high-grade ethanol (96%) for preservation.

### DNA isolation

Of each sample, we took 180–200 µg for DNA extraction, using the entire sample if less material was available (minimum: 21 µg). We extracted the DNA from the fecal samples using a QIAamp^®^ Fast DNA Stool Kit Mini (Qiagen GmbH, Düsseldorf, Germany), by mostly following the provided protocol. However, to ensure proper homogenization, we added 2–3 bashing beads (ZR Bashing Bead™ 2.0 mm, Zymo Research, Irvine, CA, USA) and applied the Disruptor Genie™ (Scientific Industries SI™, Bohemia, NY, USA). Additionally, we increased the incubation time in Buffer AL and proteinase K from 10 to 30 min. Throughout the entire process, including isolation, we included two negative extraction controls, i.e., empty vials, along with the faecal samples. We used a NanoDrop2000c UV-Vis spectrophotometer (NanoDrop Technologies, Wilmington, DE, USA) to determined DNA quality and quantity, diluting samples to 20 ng/µL if the final DNA concentration was higher than 100 ng/µL. Of the original 220 fresh scat samples, we obtained quality DNA in 178 of them, which were used in following steps (January, 19 samples; March, 18; April, 16; May, 14; June, 16; July, 12; August, 14; September, 16; October, 17; November, 18; December, 18).

### Construction of sequencing library

We constructed a NGS sequence library by means of an amplicon PCR, followed by an indexing PCR, as in previous studies (Xavier et al. 2018; Masello et al. 2021, 2023; Schumm et al. 2023; Austad et al. 2025). In this case, we used two primers: one targeting Bilateralia, and a second one amplifying the second internal transcribed spacer of nuclear ribosomal DNA (ITS2) of plant nuclear DNA (Table 1). To both primers, we attached Illumina overhang adapters (P5 for forward primers: 5′-TCGTCGGCAGCGTCAGATGTGTATAAGAGACAG-3′ and P7 for reverse primers: 5′-GTCTCGTGGGCTCGGAGATGTGTATAAGAGACAG-3′). We included PCR-grade water as a negative control in all PCR runs, as well as negative extraction controls and positive controls (DNA isolated from plants). We used QIAxcel Advanced (QIAGEN) high-resolution capillary gel electrophoresis to visualize the PCR amplicons.

**Table 1:** List of primers used for the detection of molecular operational taxonomic units in fecal samples from Burrowing Parrot *Cyanoliseus patagonus* from El Cóndor, north-eastern Patagonia, Argentina

Prey target	Gene	Primer name	Sequence 5′-3′	Annealing temperature (°C)	Amplicon size (bp)	Reference
Bilateralia	*nuclear SSU* *rDNA*	BilSSU1100_f	AGAGGTGAAATTSTTGGAYCG	60	∼245	Jarman et al. 2004
		BilSSU1300_r	CCTTTAAGTTTCAGCTTTGCA			
Plant	*ITS2*	UniPlantF	TGTGAATTGCARRATYCMG	56	187–387 bp	Moorhouse-Gann et al. 2018
		UniPlantR	CCCGHYTGAYYTGRGGTCDC			

Next, using the Illustra™ ExoproStar 1-Step Kit for enzymatic PCR clean-up (GE Healthcare, UK) and following the provided protocol, we purified a 5 µL aliquot of the amplicon PCR. Following this, we performed an index PCR to individually mark each PCR product with specific Illumina indices added to the P5 and P7 overhang adapters. Subsequently, we purified and normalized the index PCR products using the SequalPrep™ Normalization Plate Kit (Thermo Fisher Scientific, USA, Waltham, MA, ). In the final step, we pooled 2 µL of each normalized and individually tagged sample. We sent the samples to SEQ-IT GmbH & Co. KG, Kaiserslautern, Germany, for sequencing, using 250 bp paired-end reads on a MiSeq desktop sequencer (Illumina, San Diego, CA, USA).

### Molecular operational taxonomic units

**Fig. 1:**
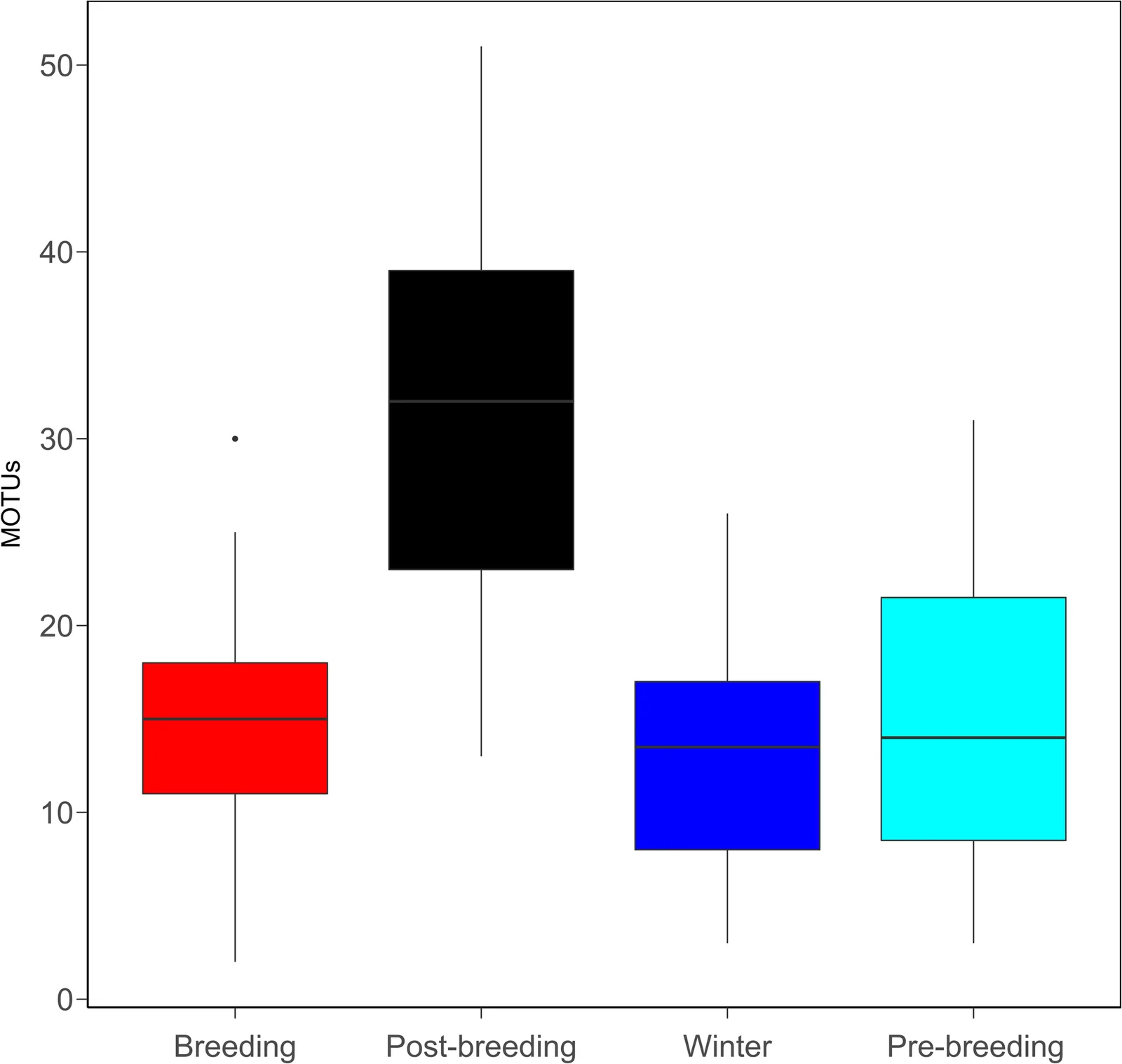
The number of plant (class Spermatopsida) molecular operational taxonomic units (MOTUs) per sample. The samples correspond to fresh fecal samples from Burrowing Parrot *Cyanoliseus patagonus* roosting in the village of El Cóndor, north-eastern Patagonia, Argentina

**Fig. 2:**
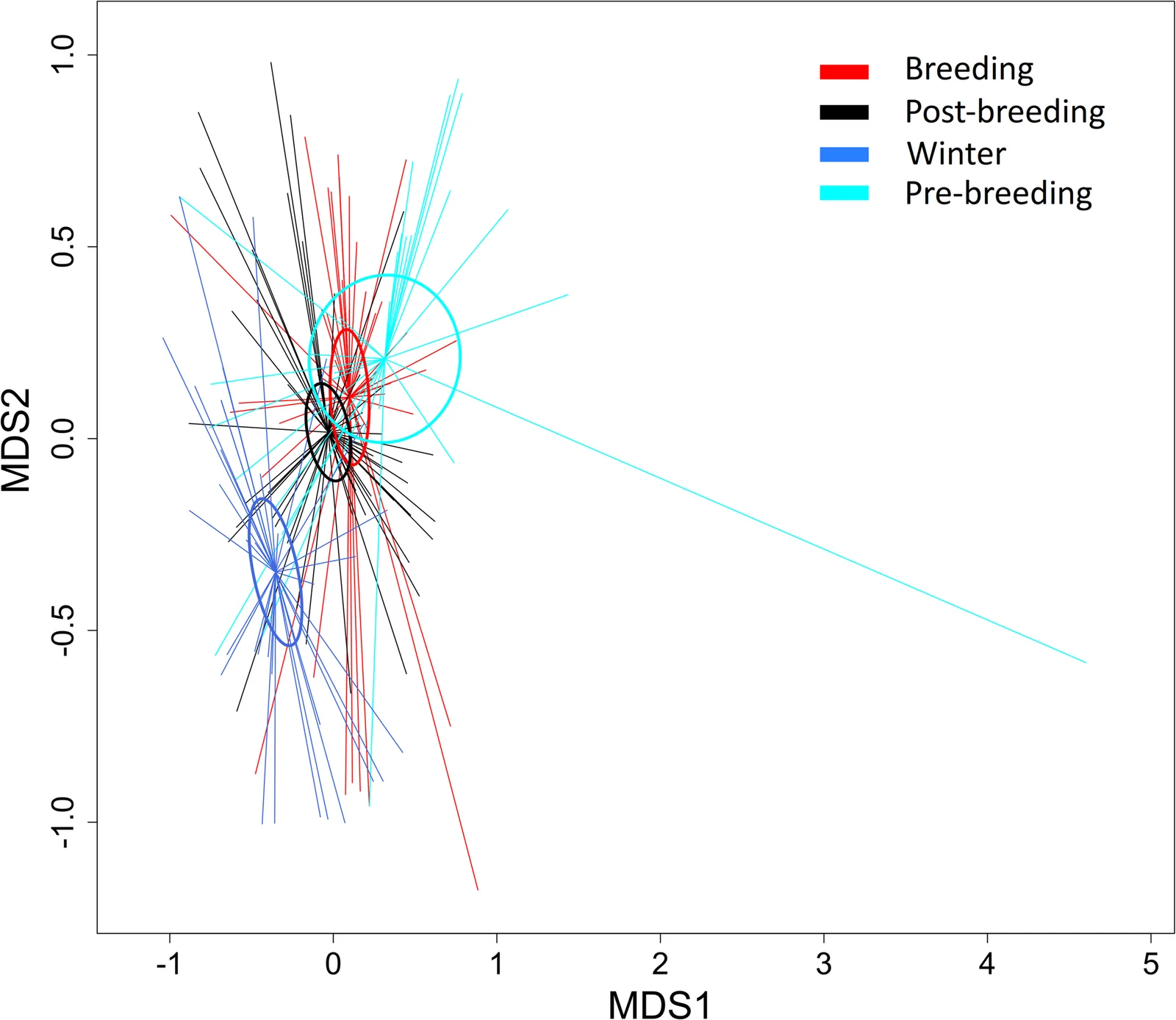
Species level diet composition of the Burrowing Parrot *Cyanoliseus patagonus* from El Cóndor in north-eastern Patagonia, Argentina. The data correspond to plant (class Spermatopsida) molecular operational taxonomic units (MOTUs) identified at the species level across the main life cycle stages (breeding, post-breeding, winter, pre-breeding). MDS1 and MDS2 are the two dimensions into which the multidimensional data were collapsed using non-metric multidimensional scaling (NMDS). We included NMDS ellipses to accentuate the corresponding centroids of the four life cycle stages, and NMDS lines to connect each sample to its respective centroid

**Fig. 3:**
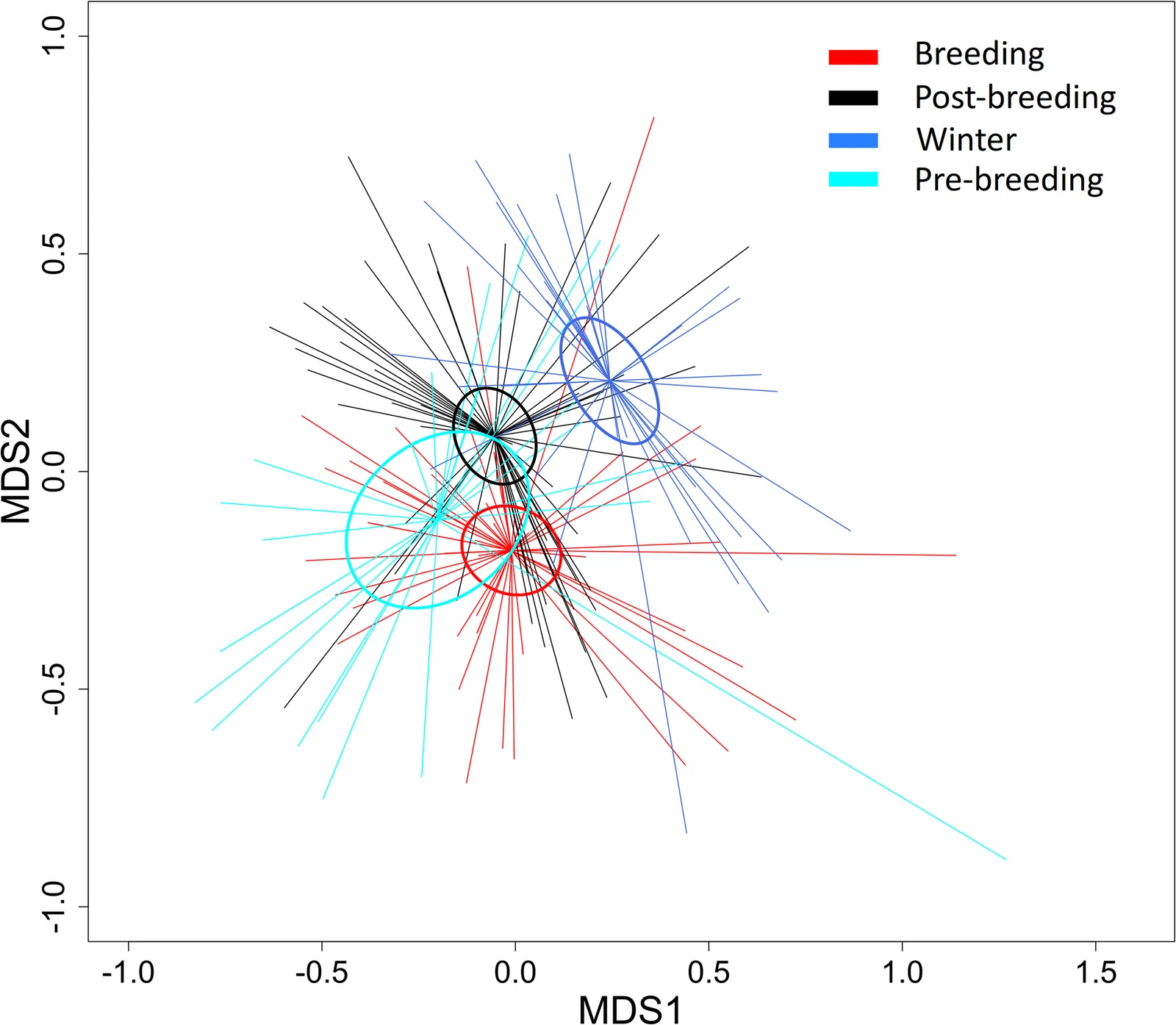
Genus level diet composition of the Burrowing Parrot *Cyanoliseus patagonus* from El Cóndor in north-eastern Patagonia, Argentina. The data correspond to plant (class Spermatopsida) molecular operational taxonomic units (MOTUs) identified at the genus level across the main life cycle stages (breeding, post-breeding, winter, pre-breeding). MDS1 and MDS2 are the two dimensions into which the multidimensional data were collapsed using non-metric multidimensional scaling (NMDS). We included NMDS ellipses to accentuate the corresponding centroids of the four life cycle stages, and NMDS lines to connect each sample to its respective centroid

**Fig. 4:**
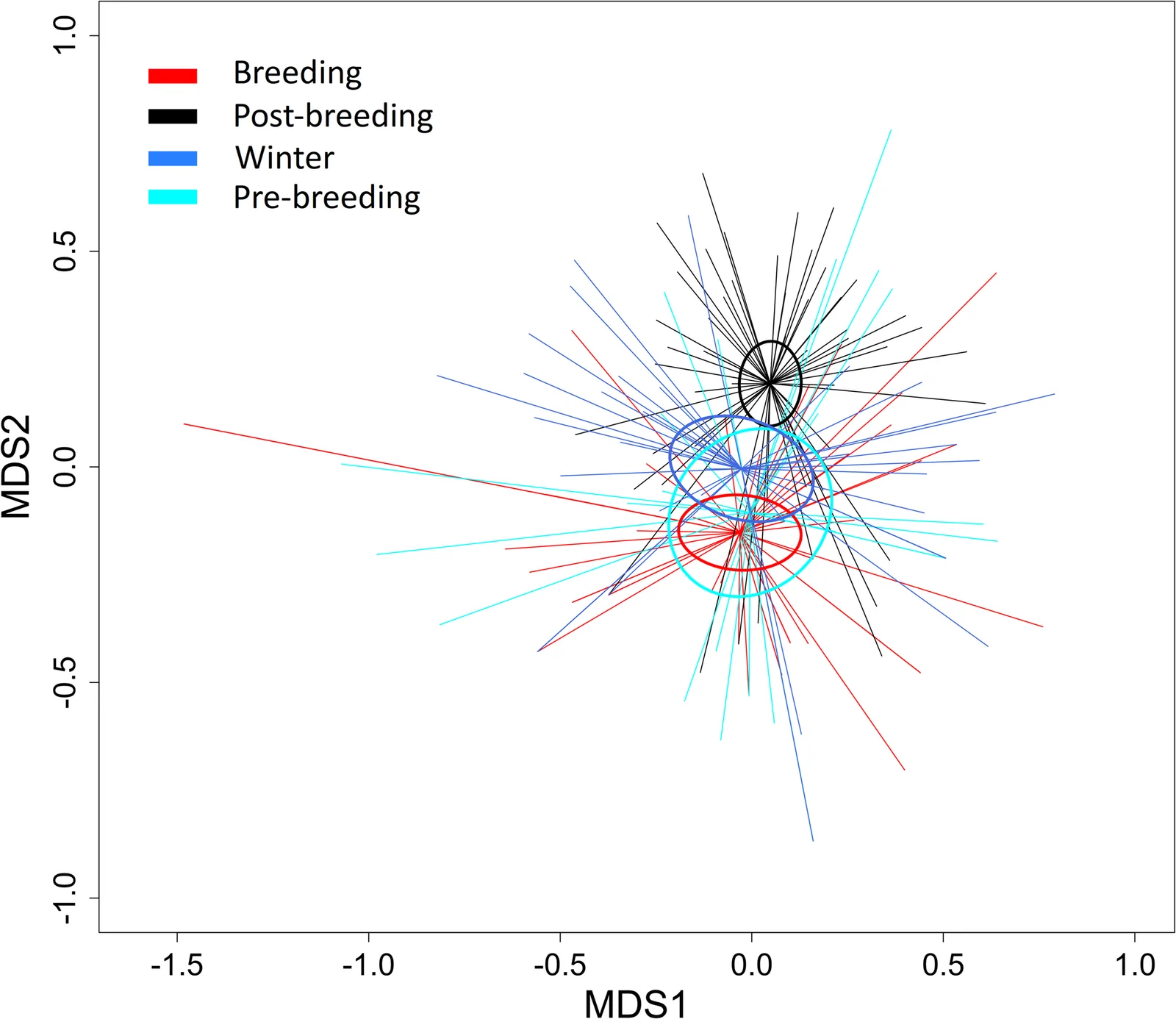
Family level diet composition of the Burrowing Parrot *Cyanoliseus patagonus* from El Cóndor in north-eastern Patagonia, Argentina. The data correspond to plant (class Spermatopsida) molecular operational taxonomic units (MOTUs) identified at the family level across the main life cycle stages (breeding, post-breeding, winter, pre-breeding). MDS1 and MDS2 are the two dimensions into which the multidimensional data were collapsed using non-metric multidimensional scaling (NMDS). We included NMDS ellipses to accentuate the corresponding centroids of the four life cycle stages, and NMDS lines to connect each sample to its respective centroid

We performed the following analyses to produce a list of molecular operational taxonomic units (MOTUs) from the raw Illumina sequence data: (1) sequence quality assessment with FASTQC (Andrews 2010), (2) adapter removal and quality trimming of paired-end reads with TRIMMOMATIC (minimum quality score of 20 over a sliding window of 4 bp; Bolger et al. 2014), (3) merging of overlapping paired-end reads with FLASH (Magoč et al. 2011), (4) conversion of sequences to FASTA format with FASTX-Toolkit (Hannon 2010), (5) amplicon extraction with MOTHUR (Schloss et al. 2009), (6) removal of identical replicates (dereplicate; derep_fulllength), detection and removal of chimeric sequences (uchime_denovo), and sequence clustering into molecular MOTUs with USEARCH (Edgar 2010), (7) matching MOTU sequences to reference sequences in the National Center for Biotechnology Information (NCBI) GenBank nucleotide database, using a minimum sequence identity of 90% and a maximum e-value of 0.00001, with the BLASTn algorithm (Altschul et al. 1990). We performed all these analyses using a custom workflow in GALAXY (Afgan et al. 2018). For the taxonomic assignment, we used the percentage similarity of the query and the reference sequences. Given that short fragments are less likely to convene reliable taxonomic information, we only retained sequences that were at least 190 base pairs long, and showed a BLASTn assignment match > 98% (Deagle et al. 2009; Masello et al. 2021, 2023; Schumm et al. 2023; Austad et al. 2025; Tables S1 and S2). As in previous studies (Masello et al. 2021, 2023; Schumm et al. 2023; Austad et al. 2025), we assigned MOTUs to the species level when the BLASTn assignment match was > 99.5%, and all of the retained hits of a MOTU corresponded to the same species and matched the same quality criteria (sequence identity, sequence length, e-value, bit-score; Tables S1 and S2). Otherwise, we assigned the MOTU to the lowest shared taxonomic level (Genus, Family, Order, Class; Figs 1, 2, 3 and 4). Negative controls were included and did not show any contamination.

### Sample size, data processing and statistical analyses

Previous studies of the Burrowing Parrot at El Cóndor (Masello and Quillfeldt 2002, 2003, 2004), revealed four main stages in the life cycle of this species. The ‘breeding season’ (hereafter, breeding) spans from October to December. The ‘post-breeding season’ (hereafter, post-breeding) is the time between the juveniles’ fledging and their independence from their parents (January to April). The ‘winter season’ (hereafter, winter) encompasses the time the pair is neither caring for juveniles nor preparing for the next breeding season (May to July). The ‘pre-breeding season’ (hereafter, pre-breeding) is characterized by nest preparation and cleaning prior to the breeding season (August to September). Thus, to investigate potential differences in diet across these main life cycle stages, we split the samples into the following groups: breeding (53 samples), post-breeding (53 samples), winter (42 samples), and pre-breeding (30 samples). For the analyses, we used presence/absence data of each MOTU, and calculated their frequency of occurrence per life cycle stage. First, we performed non-metric multidimensional scaling (NMDS) using the *metaMDS* function in the R package VEGAN (Oksanen et al. 2012) to visualize differences in Burrowing Parrot dietary composition. Using rank orders, NMDS simplifies visualization and interpretation by collapsing information originally in multiple dimensions into two dimensions. In community ecology, it is considered the most unconstrained ordination method (Faith et al. 1987; Minchin 1987). We also used the function *metaMDS* to investigate the correspondence between the two-dimensional configuration and the original configuration. This function computes a stress parameter, with values < 0.05 denoting excellent, < 0.1 very good, and < 0.2 good correspondence. The stress values we obtained were: 0.199 for the species level, 0.261 for the genus level, and 0.258 for the family level. To enhance visualization and interpretation, we applied the function *ordispider*, which connects each sample to the centroid (defined as the arithmetic mean position of all the points) of the category to which it belonged. We also used the function *ordiellipse* to visually accentuate the centroids corresponding to the four life cycle stages (Oksanen et al. 2012). In addition, we ran a permutational multivariate analysis of variance based on distance matrices (PERMANOVA), using the function *adonis*, and we tested for the multivariate homogeneity of group dispersions (variances) using the function *betadisper*.

We labeled plant MOTUs into broad categories according to their likely source: native, introduced, presumed introduced, cultivated, ornamental, and undetermined (Table 2), to evaluate differences in plant consumption among the groups. To do so, we used information available in three databases: Flora Argentina (Anton and Zuloaga 2025), the Global Biodiversity Information Facility (GBIF 2025), and Plants of the World Online (Plants of the World Online 2025). More specifically, to distinguish between native and introduced plants, we used information provided in the distribution maps in Plants of the World Online (2025), complemented with information available at Global Biodiversity Information Facility (2025). Based on Richardson et al. (2000), we labeled MOTUs as ‘introduced’ in cases of plants that have been accidentally or deliberately introduced to the area by humans, excluding plants that exist solely under cultivation/planting without self-reproducing populations (labeled ‘cultivated’). We labeled MOTUs as ‘presumed introduced’ when Plants of the World Online (2025) does not record the plant species as introduced to the studied area, yet it is known to have been introduced elsewhere in Argentina. As ‘ornamental’ MOTUs we labeled those plants that people keep in the gardens of the village of El Cóndor but are not found in the surrounding scrubland and patches of Monte vegetation. We used the category ‘undetermined’ when the taxonomic level did not allow for clear labeling. Plant taxonomic levels follow those in the Flora Argentina database (Anton and Zuloaga 2025). For Bilateralia, we used the following ecological categories: oral cavity parasite, intestinal parasite, ingested ectoparasite, presumed ingested ectoparasite, bycatch, and presumed bycatch (Table 3). We classified MOTUs as ‘ingested ectoparasite’ in cases when ectoparasites of the same taxonomic rank are known to affect Burrowing Parrots (Di Iorio et al. 2010). We classified MOTUs as “presumed ingested ectoparasites” when the organism’s lifestyle allows to suspect this, but we lacked confirmation from the literature. We used the category ‘bycatch’ in cases when organisms of the same taxonomic rank are known to be accidentally ingested by other birds with their food or water e.g. organisms present in soil or water (Brodie 1976; Carta et al. 2020; Burke et al. 2025). We labeled MOTUs with ‘potential bycatch’ when the organism’s typical habitat allows to infer the possibility of accidental ingestion, but we could not find previous records in the literature. Bilateralia taxonomic levels follow those used in Global Biodiversity Information Facility (2025).

**Table 2:** Valid molecular operational taxonomic units (MOTUs) of plants belonging to the class Spermatopsida present in the diet of the Burrowing Parrot *Cyanoliseus patagonus* from El Cóndor, north-eastern Patagonia, Argentina, and frequency of occurrence per life cycle stage (% of samples). MOTUs are ordered by the taxonomic ranks order and family (marked bold). Where available, the plant species’ phenology phases in north-eastern Patagonia corresponding to the parrot life cycle stages are indicated with superscripts: leaf stage (L), flowering development (FL) and fruit growth and seed release (FR). See also Fig. 5 for temporal correspondence between plants identified in the parrot’s diet and plant phenology across the life-cycle stages of Burrowing Parrots from El Cóndor

MOTUs	English common name	Spanish common name	Category	Frequency of occurrence (% of samples)			
				Breeding	Post-breeding	Winter	Pre-breeding
Ephedrales							
	**Ephedraceae**						
Ephedraceae	Jointfir	Efedráceas	Undetermined	1.9 ^FL^	0 ^FR^	0	0
Pinales							
	**Pinaceae**						
Pinaceae	Pine Family	Pináceas	Undetermined	24.1 ^L−FL−FR^	50.9 ^L^	18.6 ^L^	23.3 ^L−FL^
*Picea glauca*	Alberta Spruce	Pícea blanca	Ornamental	3.7 ^L−FL^	32.1 ^L−FR^	14 ^L^	0 ^L^
*Pinus* sp.	Pine	Pinos	Introduced	1.9 ^L−FL^	1.9 ^L−FL−FR^	0 ^L^	10 ^L^
*Pinus sylvestris*	Scotch Pine	Pino	Ornamental	14.8 ^LFL^	24.5 ^L−FL−FR^	4.7 ^L^	13.3 ^L^
	**Taxaceae**						
*Taxus canadensis*	American Yew	Tejo común, tejo negro	Ornamental	0 ^L^	1.9 ^L^	0 ^L^	0 ^L−FL^
	**Cupressaceae**						
*Cupressus sempervirens*	Italian Cypress	Ciprés común	Ornamental	1.9 ^L−FL^	1.9 ^L−FR^	0 ^L−FR^	3.3 ^L−FL^
*Juniperus communis*	Common Juniper	Enebro	Ornamental	0 ^L−FL^	1.9 ^L−FR^	0 ^L−FR^	0 ^L−FR^
Poales							
	**Cyperaceae**						
Cyperaceae	Sedge	Ciperáceas	Undetermined	11.1 ^L−FL−FR^	1.9 ^L−FR^	7 ^L^	0 ^L−FL^
*Cladium* sp.	Saw-grass	-	Undetermined	1.9 ^L−FL^	1.9 ^L−FR^	0	0 ^L^
	**Poaceae**						
*Alopecurus* sp.	Foxtail	Colas de zorro	Undetermined	0 ^L−FL^	5.7 ^L−FR^	2.3 ^FR^	0 ^L^
*Alopecurus myosuroides*	Black-grass	Pasto negro europeo	Introduced	0 ^L−FL−FR^	1.9 ^L−FR^	2.3 ^L^	0 ^L−FL^
*Amelichloa* sp.	Needle Grass	Pajas vizcacheras	Native	0 ^L−FL−FR^	1.9 ^L−FR^	14 ^L^	3.3 ^L^
*Amelichloa caudata*	Chilean Ricegrass	Paja vizcachera	Native	3.7 ^L−FL−FR^	1.9 ^L−FR^	9.3 ^L^	3.3 ^L^
*Anthoxanthum* sp.	Vernal Grass	Hierbas olorosas	Introduced	0 ^L−FL^	1.9 ^L−FR^	0	0 ^L^
*Arrhenatherum* sp.	Oatgrass	Avenas falsas	Introduced	0 ^L−FL^	1.9 ^L−FR^	0	0 ^L^
*Arrhenatherum elatius*	Bulbous Oatgrass	Fromental, pasto cebolla	Introduced	0 ^L−FL^	3.8 ^L−FR^	0	0 ^L^
*Avena* sp.	Oat	Avenas	Introduced	53.7 ^L−FL−FR^	56.6 ^FR^	27.9 ^L^	16.7 ^L^
*Avena sativa*	Algerian Oat	Avena bandera, avena chilena, avena desnuda, avena blanca	Cultivated	50 ^L−FL−FR^	56.6 ^FR^	30.2 ^L^	16.7 ^L^
*Avenella flexuosa*	Wavy Hair-grass	Heno, heno común	Undetermined	0 ^L−FL^	1.9 ^FR^	0 ^L^	0 ^L^
*Bromus catharticus*	Rescue Brome	Cebadilla criolla, cebadilla australiana	Native	0 ^L−FL^	1.9 ^FR^	0 ^L^	0 ^L^
*Cenchrus* sp.	Fountaingrass	Zacates	Undetermined	0 ^L^	0 ^L−FL−FR^	4.7 ^FR^	0 ^L^
*Cenchrus echinatus*	Southern Sandbur, Spiny Sandbur, Mossman River Grass	Zacate cadillo, guizacillo de Cuba, ojo de hormiga, huachapore	Undetermined	1.9 ^L^	3.8 ^L−FL−FR^	7 ^FR^	0 ^L^
*Cynodon* sp.	Cynodon	Gramillas	Introduced	1.9 ^L^	45.3 ^L−FL−FR^	2.3 ^FR^	3.3 ^L^
*Cynodon dactylon*	Bermuda Grass	Gramillón, gramilla brava	Introduced	0 ^L−FL^	17 ^L−FR^	0	3.3 ^L^
*Cynodon nlemfuensis*	African Bermuda Grass	Pasto estrella, tumbabobos	Introduced	0 ^L−FL^	34 ^L−FL−FR^	0	3.3 ^L^
*Dactylis* sp.	Orchardgrass	Pastos ovillo	Introduced	0 ^L−FL−FR^	7.5 ^L−FR^	2.3 ^L^	0 ^L^
*Dactylis glomerata*	Barnyard Grass	Pasto ovillo	Introduced	0 ^L−FL^	7.5 ^L−FL−FR^	2.3 ^L^	0 ^FL^
*Eleusine tristachya*	American Yard-grass	Grama carraspera	Native	1.9 ^L^	5.7 ^L−FL−FR^	0 ^FR^	0 ^L^
*Elytrigia repens*	Quack Grass	Grama	Introduced	0 ^L−FL^	1.9 ^L−FR^	0 ^L^	0 ^L^
*Eragrostis mexicana*	Mexican Lovegrass	Pasto hediondo	Native	0 ^L−FL^	1.9 ^L−FR^	0	0 ^L^
*Eriocoma* sp.	Needlegrass	Arroz de indio	Introduced	0 ^L−FL−FR^	0 ^L−FR^	2.3 ^L^	0 ^L^
*Festuca* sp.	Fescue	Festucas	Native	3.7 ^L−FL^	11.3 ^L−FL−FR^	0 ^L−FR^	0 ^L^
*Holcus* sp.	Soft-grasses	Pastos dulces, pastos miel	Introduced	0 ^L^	0 ^L−FL−FR^	2.3 ^L^	0 ^L^
*Holcus lanatus*	Common Velvetgrass	Heno blanco, pasto dulce, pasto lanudo, pasto miel	Introduced	0 ^L^	9.4 ^L−FL−FR^	4.7 ^L^	0 ^L^
*Hordeum* sp.	Barleys	Colas de zorro	Undetermined	16.7 ^L−FL^	43.4 ^FR^	20.9 ^L^	36.7 ^L^
*Hordeum murinum*	Mouse Barley	Cola de zorro	Introduced	3.7 ^L−FL−FR^	5.7 ^FR^	0 ^L^	0 ^FL^
*Hordeum vulgare*	Barley	Cebada forrajera	Cultivated	16.7 ^L−FL−FR^	60.4 ^FR^	41.9 ^L^	30 ^L^
*Lolium* sp.	Darnel, Ryegrass	Lolium, raigrás	Introduced	0 ^FL^	18.9 ^FR^	7 ^L^	0 ^L^
*Lolium perenne*	English Ryegrass	Raigrás	Introduced	7.4 ^L−FL−FR^	24.5 ^L^	9.3 ^L^	0 ^L^
*Milium effusum*	Millet Grass	Mijo silvestre	Presumed introduced	0 ^L−FL^	1.9 ^L−FL−FR^	0 ^L^	0 ^L^
*Molinia* sp.	Moorgrass	Pastos morados, molinias	Presumed introduced	9.3 ^L^	11.3 ^L−FL−FR^	16.3 ^FR^	6.7 ^L^
*Nassella* sp.	Tussockgrass	Flechillas	Native	0 ^L−FL^	0 ^L−FR^	2.3 ^L^	0 ^L^
*Poa* sp.	Bluegrass, Meadow Grass	Poas	Undetermined	0 ^L−FL−FR^	1.9 ^L−FR^	0 ^L^	0 ^L^
*Poa infirma*	Early Meadow-grass	Pastito de invierno	Introduced	3.7 ^L−FL−FR^	1.9 ^FR^	0	3.3 ^L^
*Poa pratensis*	English Meadow Grass	Poa de los prados, pasto mallín	Introduced	1.9 ^L−FL^	15.1 ^FR^	0	0 ^L^
*Poa trivialis*	Rough Bluegrass	Poa común, gamilla	Introduced	3.7 ^L−FL^	0 ^L−FL−FR^	0	0 ^L^
*Poaceae*	Grass	Gramíneas	Undetermined	83.3 ^L−FL−FR^	100 ^L−FL−FR^	86 ^L−FL−FR^	76.7 ^L−FL−FR^
*Schismus* sp.	Mediterranean Grass	Pastitos de invierno	Introduced	0 ^FL−FR^	0	2.3 ^L^	0 ^L^
*Sorghum bicolor*	Giant-millet	Sorgo, mijo grande	Cultivated	0 ^L−FL−FR^	1.9 ^FR^	0	0 ^L^
*Thinopyrum* sp.	Wheatgrass	Agropiros	Introduced	0 ^L^	1.9 ^L−FL−FR^	0 ^L−FR^	0 ^L^
*Trisetaria* sp.	-	Trisetarias	Presumed introduced	0 ^L−FL^	1.9 ^L−FL−FR^	0 ^L^	0 ^L^
*Trisetopsis* sp.	-	Pasto	Presumed introduced	0 ^L−FL^	1.9 ^L−FL−FR^	0 ^L^	0 ^L^
*Trisetum* sp.	Oatgrass	Pasto	Presumed introduced	0 ^L−FL^	11.3 ^L−FL−FR^	0 ^L^	0 ^L^
*Triticum* sp.	Wheat	Trigos	Cultivated	18.5 ^L−FL−FR^	28.3 ^FR^	11.6 ^L^	13.3 ^L^
*Triticum aestivum*	Bread Wheat	Trigo del pan, trigo blando	Cultivated	40.7 ^L−FL−FR^	54.7 ^FR^	37.2 ^L^	33.3 ^L^
*Triticum monococcum*	Einkorn	Escanda menor	Cultivated	1.9 ^L−FL−FR^	7.5 ^FR^	7 ^L^	3.3 ^L^
Ranunculales							
	**Ranunculaceae**						
Ranunculaceae	Buttercup Family	Ranunculáceas	Undetermined	0 ^L−FL−FR^	5.7 ^L−FR^	2.3 ^L^	10 ^L^
Proteales							
	**Platanaceae**						
*Platanus* x *hispanica*	London Plane	Plátano	Ornamental	0 ^FL^	3.8 ^FR^	2.3 ^FR^	3.3 ^FR−L^
	**Proteaceae**						
Proteaceae	Proteas	Proteáceas	Undetermined	1.9 ^FL^	0 ^FR^	0	0 ^FL^
Caryophyllales							
	**Tamariscaceae**						
*Tamarix* sp.	Tamarisk	Tamariscos	Introduced	0 ^L−FL^	1.9 ^L−FR^	0	0
*Tamarix gallica*	French Tamarisk	Tamarisco, taray, taray de Europa	Introduced	0 ^L−FL^	1.9 ^L−FR^	0	0
	**Plumbaginaceae**						
Plumbaginaceae	Leadwort	Plumbagináceas	Ornamental	0 ^L−FL^	0 ^L−FL−FR^	0	3.3 ^L−FL^
	**Polygonaceae**						
Polygonaceae	Buckwheat	Centinodias	Undetermined	0 ^L^	1.9 ^L−FL−FR^	0	0 ^L^
*Rumex* sp.	Dock	Lengua de vaca	Native	0 ^L−FL^	1.9 ^L−FR^	0	0 ^L^
	**Amaranthaceae**						
Amaranthaceae	Pigweed	Amarantos	Undetermined	16.7 ^L−FL^	45.3 ^L−FL−FR^	14 ^FR^	13.3 ^L^
*Amaranthus deflexus*	Argentine Amaranth	Bledo rastrero	Native	0 ^L^	1.9 ^L−FL−FR^	0	0 ^L^
*Amaranthus palmeri*	Carelessweed	Quintonil tropical	Introduced	0 ^L^	1.9 ^L−FL−FR^	0	0 ^L^
*Atriplex* sp.	Oraches	Plantas de sal, abanicos	Undetermined	0 ^L−FL−FR^	1.9 ^L−FL−FR^	0 ^L^	0 ^L^
*Atriplex micrantha*	Russian Atriplex	Cachiyuyo	Introduced	0 ^L−FL^	1.9 ^L−FL−FR^	0 ^L^	0 ^L^
*Atriplex semibaccata*	Australian Saltbush	Cachiyuyo	Introduced	5.6 ^L−FL^	3.8 ^L−FR^	4.7 ^L^	0 ^L^
*Atriplex undulata*	Wavy-leaved Saltbush	Cola de zorro	Native	0 ^L^	3.8 ^L−FL−FR^	0 ^L^	0 ^L^
*Bassia scoparia*	Belvedere	Alfalfa criolla	Introduced	0 ^L−FL^	11.3 ^L−FR^	0	0 ^L^
*Beta vulgaris*	Beet	Acelgas, remolachas	Cultivated	0 ^L−FL−FR^	11.3 ^L^	0 ^L^	0 ^L^
*Chenopodium* sp.	Goosefoot	Quinoas	Undetermined	0 ^L−FL^	1.9 ^L−FL−FR^	0	0 ^L^
*Chenopodium album*	Common Lambsquarters	Granasche, quinoa, yuyo blanco, yuyo colorado, quinguilla	Introduced	0 ^L−FL^	11.3 ^L−FR^	0	0 ^L^
*Dysphania* sp.	Goosefoot	Paicos	Undetermined	0 ^L−FL^	3.8 ^L−FR^	0	0 ^L^
*Heterostachys ritteriana*	-	Jume, jumecillo	Native	0 ^L^	1.9 ^L−FL−FR^	0 ^L^	0 ^L^
*Salicornia neei*	-	Salicornia	Native	1.9 ^L^	3.8 ^L−FL−FR^	0 ^L^	0 ^L^
*Salsola* sp.	Russian Thistle	Cardos rusos	Introduced	5.6 ^L^	9.4 ^L−FL−FR^	4.7 ^FR^	13.3 ^L^
*Salsola kali*	Common Saltwort	Cardo ruso	Introduced	1.9 ^L−FL^	7.5 ^L−FL−FR^	7 ^FR^	13.3 ^L^
*Salsola tragus*	Prickly Russian Thistle	Barilla	Introduced	5.6 ^L^	15.1 ^L−FL−FR^	2.3 ^FR^	13.3 ^L^
*Suaeda foliosa*	-	Vidriera	Presumed introduced	1.9 ^L−FL−FR^	0 ^L−FR^	0 ^L^	0 ^L^
	**Caryophyllaceae**						
*Cerastium* sp.	Chickweed	Cerastios	Undetermined	0 ^L−FL^	0 ^L−FL−FR^	0 ^L−FL−FR^	3.3 ^L^
*Cerastium fontanum*	Common Chickweed	Oreja de ratón, oreja de tierra, cerastio	Introduced	0 ^L−FL^	0 ^L−FL−FR^	0 ^L^	3.3 ^L^
*Stellaria pallida*	Lesser Chickweed	Capiquí	Introduced	0 ^L−FL−FR^	5.7 ^FR^	0 ^L^	3.3 ^L^
	**Aizoaceae**						
Aizoaceae	Dewplant Family	Aizoáceas	Undetermined	0 ^L−FL^	1.9 ^L−FR^	0 ^L^	0 ^L−FL^
	**Portulacaceae**						
Portulacaceae	Blinks	Verdolagas	Undetermined	1.9 ^L−FL−FR^	11.3 ^L^	0	0 ^L^
*Portulaca* sp.	Purslane	Verdolagas	Undetermined	0 ^L−FL−FR^	1.9 ^L^	0	0 ^L^
*Portulaca oleracea*	Akulikuli-kula	Verdolaga común	Introduced	1.9 ^L^	11.3 ^L−FL−FR^	0	0 ^L^
Saxifragales							
	**Paeoniaceae**						
*Paeonia lactiflora*	Chinese Peony	Peonía china, peonía híbrida, rosa de monte, rosa sin espinas	Ornamental	0 ^L−FL−FR^	1.9 ^L−FR^	0	0 ^L^
	**Crassulaceae**						
*Sedum confusum*	Lesser Mexican-stonecrop	Siempreviva de media montaña	Ornamental	0 ^L−FL−FR^	1.9 ^L^	0 ^L^	0 ^L^
Geraniales							
	**Geraniaceae**						
*Erodium cicutarium*	Alfilaria	Alfilerillo	Introduced	1.9 ^L−FL−FR^	9.4 ^FR^	0 ^L^	10 ^L^
*Geranium dissectum*	Cut-leaved Crane’s-bill	Alfilerillo	Introduced	0 ^L−FL−FR^	1.9 ^FR^	0 ^L^	0 ^L^
Myrtales							
	**Myrtaceae**						
*Eucalyptus* sp.	Gum	Eucaliptos	Introduced	3.7 ^L−FL^	7.5 ^L−FR^	0 ^L−FR^	0 ^L−FR^
*Eucalyptus camaldulensis*	River red Gum	Eucalipto rojo, gomero rojo, eucalipto colorado	Introduced	1.9 ^L−FL^	5.7 ^L−FR^	0 ^L−FR^	0 ^L−FR^
*Eucalyptus nicholii*	Narrow-leaf Black Peppermint	menta piperita negra de hojas angostas	Presumed introduced	3.7 ^L−FL^	0 ^L−FR^	0 ^L−FR^	0 ^L−FR^
Zygophyllales							
	**Zygophyllaceae**						
Zygophyllaceae	Caltrop	Zigofiláceas	Undetermined	46.3 ^L−FL−FR^	88.7 ^L−FL−FR^	62.8 ^L^	50 ^L^
*Tribulus* sp.	Puncture Vine	Abrojos	Introduced	40.7 ^L−FL^	84.9 ^L−FL−FR^	60.5 ^FR^	43.3 ^L^
*Tribulus terrestris*	Mexican Sandbur	Roseta francesa	Introduced	35.2 ^L−FL^	79.2 ^L−FL−FR^	46.5 ^FR^	43.3 ^L^
Malpighiales							
	**Passifloraceae**						
*Passiflora caerulea*	Blue Passionflower	Mburucuyá, pasionaria	Native	0 ^L−FL^	1.9 ^L−FL−FR^	0 ^FR^	0 ^L^
	**Salicaceae**						
*Salix* sp.	Willows	Sauces	Undetermined	0 ^L−FL−FR^	1.9 ^L^	0	0 ^FL^
*Salix matsudana*	Corkscrew Willow	Sauce llorón	Introduced	0 ^L−FR^	5.7 ^L^	0	0 ^FL^
	**Linaceae**						
*Linum bienne*	Pale Flax	Lino bravo, pálido, silvestre, bienal	Introduced	0 ^L−FL−FR^	1.9 ^L−FR^	0	0 ^L^
Fabales							
	**Fabaceae**						
Fabaceae	Legumes	Leguminosas	Undetermined	24.1 ^L−FL−FR^	49.1 ^L−FL−FR^	16.3 ^L^	26.7 ^L^
*Erythrostemon* sp.	-	Barba de chivo	Native	1.9 ^L−FL^	0 ^L−FL−FR^	0	0
*Hoffmannseggia* sp.	Rushpea	Porotillo	Native	1.9 ^L−FL^	1.9 ^L−FR^	0	0 ^L^
*Medicago polymorpha*	Black Medick	Alfalfa de secano, carretón cadillo, trébol carretilla, hualputra	Introduced	0 ^L−FL−FR^	1.9 ^FR^	0 ^L^	0 ^L^
*Medicago truncatula*	Barrel Clover	Carretón	Introduced	0 ^L−FL−FR^	3.8 ^L−FR^	0	3.3 ^L−FL^
*Robinia pseudoacacia*	Black Locust	Acacia blanca	Introduced	0 ^L−FL^	5.7 ^L^	0	0 ^L^
*Senna* sp.	Sennas	Senas	Undetermined	0 ^L−FL−FR^	1.9 ^L−FL−FR^	0	0 ^L^
*Styphnolobium* sp.	-	Sóforas	Ornamental	0 ^L^	1.9 ^L−FL−FR^	0	0 ^L^
*Styphnolobium japonicum*	Chinese Scholar Tree	Sófora	Ornamental	0 ^L−FR^	1.9 ^L−FL−FR^	0 ^L−FR^	0 ^L−FR^
*Trifolium repens*	Dutch Clover	Trébol blanco	Introduced	0 ^L−FL^	3.8 ^L−FL−FR^	0 ^L^	0 ^L^
*Wisteria* sp.	Wisteria	Glicinas, glicinias	Ornamental	1.9 ^L−FL−FR^	0 ^L−FR^	0	0 ^L−FL^
Rosales							
	**Rosaceae**						
Rosaceae	Rose Family	Rosáceas	Undetermined	9.3 ^L−FL−FR^	20.8 ^L−FR^	2.3 ^FR^	10 ^L−FL^
*Prunus* sp.	Stonefruit		Undetermined	3.7 ^L−FL−FR^	11.3 ^L−FR^	0	0 ^L−FL^
*Rosa* sp.	Rose	Rosas	Ornamental	1.9 ^L−FL^	0 ^L−FR^	0	0 ^L^
*Rosa banksiae*	Lady Banks’ Rose, Tombstone Rose, Banks’ Rose	-	Ornamental	0 ^L−FL^	1.9 ^L^	0	0 ^L^
*Rosa chinensis*	Bengal Rose	Rosa china	Ornamental	1.9 ^L−FL^	0 ^L^	0	0 ^L^
	**Rhamnaceae**						
Rhamnaceae	Buckthorn Family	-	Native	1.9 ^L−FL^	11.3 ^L−FR^	4.7 ^L^	3.3 ^L^
*Condalia* sp.	Snakewood	Piquillín	Native	0 ^L−FL^	3.8 ^L−FR^	7 ^L^	0 ^L^
	**Ulmaceae**						
Ulmaceae	Elm Family	Ulmáceas	Undetermined	0 ^L−FL−FR^	15.1 ^L^	0	0 ^L^
*Ulmus* sp.	Elm	Olmos	Introduced	0 ^L−FL−FR^	15.1 ^L^	0	0 ^L^
*Ulmus pumila*	Chinese Elm	Olmo de Siberia	Introduced	0 ^L−FL−FR^	7.5 ^L^	0	0 ^L^
	**Cannabaceae**						
*Humulus lupulus*	Bine	Lúpulo	Cultivated	0 ^L^	0 ^L−FL−FR^	2.3 ^FR^	3.3 ^L^
	**Moraceae**						
Moraceae	Mulberries	Moráceas	Undetermined	0 ^L−FL^	0 ^L−FR^	0	10 ^L^
*Morus alba*	Mulberry	Morera blanca	Introduced	0 ^L−FL^	0 ^L−FR^	0	10 ^L^
	**Urticaceae**						
Urticaceae	Nettle Family	Ortigas	Undetermined	0 ^L−FL−FR^	0 ^L−FL−FR^	7 ^FR^	0 ^L^
Cucurbitales							
	**Cucurbitaceae**						
Cucurbitaceae	Gourds	Cucurbitáceas	Undetermined	31.5 ^L−FL−FR^	41.5 ^L−FL−FR^	48.8 ^FR^	26.7 ^L^
*Cucumis* sp.	Cucumber	Melones	Introduced	9.3 ^L−FL^	18.9 ^L−FL−FR^	27.9 ^FR^	23.3 ^L^
*Cucurbita* sp.	Gourds	Zapallos y calabazas	Undetermined	9.3 ^L−FL^	5.7 ^L−FR^	4.7 ^FR^	6.7 ^L^
Fagales							
	**Fagaceae**						
*Fagus orientalis*	Oriental Beech	Haya oriental, haya del Asia Menor	Ornamental	0 ^L−FL^	13.2 ^L−FR^	0	3.3 ^L^
*Glycine max*	Soybean, Soy Bean, Soya Bean	Soja	Cultivated	0 ^L−FL^	7.5 ^L−FR^	0	0 ^L^
*Quercus robur*	English Oak	Roble	Ornamental	0 ^L−FL^	3.8 ^L−FR^	0	0 ^L^
	**Juglandaceae**						
Juglandaceae	Walnut Family	Juglandáceas	Undetermined	0 ^L−FL^	1.9 ^L−FR^	0	0 ^L^
*Juglans* sp.	Nogal	Nogales	Undetermined	0 ^L−FL−FR^	1.9 ^L−FR^	0	0 ^L^
*Juglans regia*	Carpathian Walnut	Nogal	Cultivated	0 ^L−FL^	1.9 ^L−FR^	0	0 ^L^
	**Betulaceae**						
Betulaceae	Alder	Betuláceas	Undetermined	1.9 ^L−FL−FR^	28.3 ^L−FR^	9.3 ^FR^	3.3 ^L^
*Betula* sp.	Birch	Abedules	Introduced	0 ^L−FL−FR^	5.7 ^L−FR^	0	0 ^L^
*Betula glandulosa*	Dwarf Birch	Abedul enano, abedul de montaña	Ornamental	0 ^L−FL^	1.9 ^L−FL−FR^	0	0 ^L^
*Betula pendula*	European Silver Birch	Abedul	Introduced	1.9 ^L−FL^	15.1 ^L−FR^	0	3.3 ^L^
*Betula pubescens*	Downy Birch	Abedul, bedul, aliso blanco, chopa blanca	Ornamental	0 ^L−FL−FR^	3.8 ^L−FR^	0	0 ^L^
*Carpinus betulus*	European Hornbeam	Carpe blanco, carpe europeo, carpe, abedulillo, hojaranzo, charmilla, ojaranzo	Ornamental	0 ^L−FL−FR^	13.2 ^L−FR^	7 ^FR^	0 ^L^
*Carpinus laxiflora*	Loose-flower Hornbeam	Carpino de flores laxas	Ornamental	0 ^L−FL−FR^	3.8 ^L−FR^	2.3 ^FR^	0 ^L^
Brassicales							
	**Brassicaceae**						
Brassicaceae	Cabbage Family	Crucíferas	Undetermined	24.1 ^L−FL−FR^	66 ^FR^	37.2 ^L^	16.7 ^L^
*Brassica* sp.	Brassicas	Brassicas	Introduced	1.9 ^L−FL−FR^	3.8 ^FR^	4.7 ^L^	0 ^L^
*Brassica oleracea*	Cabbage	Col silvestre	Introduced	0 ^L−FL−FR^	15.1 ^FR^	14 ^L^	6.7 ^L^
*Brassica rapa*	Bird’s Rape	Nabo silvestre, yuyo	Introduced	9.3 ^L−FL−FR^	18.9 ^FR^	20.9 ^L^	3.3 ^L^
*Diplotaxis* sp.	Wall-rockets	Jaramagos	Introduced	13 ^L−FL^	37.7 ^FR^	7 ^L^	3.3 ^L^
*Eruca* sp.	Rocketsalad	Rúculas	Introduced	1.9	5.7	0 ^L^	3.3 ^L^
*Raphanus* sp.	Radish	Rábanos salvajes	Introduced	0 ^L−FL^	7.5 ^L−FL−FR^	0 ^FR^	3.3 ^L^
*Sisymbrium* sp.	Hedgemustard	Mostacillas	Introduced	0 ^L−FL−FR^	1.9 ^FR^	0 ^L^	0 ^L^
Malvales							
	**Malvaceae**						
Malvaceae	Mallow Family	Malváceas	Undetermined	0 ^L−FL−FR^	22.6 ^L−FL−FR^	2.3 ^L^	16.7 ^L^
*Malva* sp.	Cheeseweed	Malvas	Introduced	0 ^L−FL−FR^	7.5 ^L−FR^	0 ^L^	6.7 ^L^
*Tilia* sp.	Basswood	Tilos	Ornamental	0 ^L−FL−FR^	1.9 ^L−FR^	0	0 ^L^
*Tilia cordata*	Linden	Tilo	Ornamental	0 ^L−FL−FR^	5.7 ^L−FR^	0	0 ^L^
Sapindales							
	**Anacardiaceae**						
*Schinus* sp.	Peppertree	Pimenteros	Native	0 ^L−FL^	1.9 ^L−FR^	0 ^L^	0 ^L−FL^
*Schinus molle*	American Pepper	Gualeguay, molle, aguaribay, terebinto, molle blanco, molle castilla, pimentero	Native	0 ^L−FL−FR^	24.5 ^L−FR^	7 ^L^	0 ^L^
	**Sapindaceae**						
*Acer*	Maple	Arces	Introduced	0 ^L−FL^	1.9 ^L−FR^	0 ^FR^	0 ^L−FL^
Gentianales							
	**Rubiaceae**						
Rubiaceae	Bedstraw Family	Rubiáceas	Undetermined	1.9 ^L−FL−FR^	1.9 ^L−FL−FR^	0 ^L^	0 ^L^
Lamiales							
	**Oleaceae**						
Oleaceae	Ash Family	Oleáceas	Undetermined	1.9 ^L−FL−FR^	1.9 ^L−FR^	0 ^L^	0 ^L^
*Fraxinus pennsylvanica*	Downy Ash	Fresno rojo, fresno americano	Introduced	0 ^L−FL−FR^	1.9 ^L−FR^	0	0 ^L^
	**Plantaginaceae**						
Plantaginaceae	Plantain	Plantagináceas	Undetermined	0 ^L−FL−FR^	5.7 ^L−FL−FR^	0 ^L^	3.3 ^L^
*Plantago* sp.	Fleaworts	Llantenes	Undetermined	0 ^L−FL−FR^	3.8 ^L^	0 ^L^	3.3 ^L^
	**Scrophulariaceae**						
*Myoporum laetum*	New Zealand Manatoka	Siempreverde, transparente	Introduced	0 ^L^	1.9 ^L^	0 ^L−FL^	0 ^L−FL^
Solanales							
	**Convolvulaceae**						
Convolvulaceae	−	Campanillas	Undetermined	5.6 ^L−FL−FR^	0 ^L−FL−FR^	2.3 ^FR^	0 ^L^
*Convolvulus* sp.	Bindweed	Campanitas	Undetermined	5.6 ^L^	0 ^L−FL−FR^	0	0 ^L^
*Ipomoea* sp.	Morning-glory	Campanitas	Undetermined	0 ^L−FL^	0 ^L−FL^	2.3 ^FR^	0 ^L^
*Ipomoea batatas*	Sweet Potato	Batata	Cultivated	0 ^L−FL^	0 ^L−FR^	2.3 ^FR^	0 ^L^
	**Solanaceae**						
*Solanum* sp.	Divine Nightshade	Solanáceas	Undetermined	0 ^L−FL^	0 ^L−FR^	0	3.3 ^L^
*Solanum lycopersicum*	Garden Tomato	Tomate	Cultivated	0 ^L−FL−FR^	1.9 ^L−FR^	0	0 ^L^
Apiales							
	**Pittosporaceae**						
Pittosporaceae	Pittosporum Family	Pitosporáceas	Undetermined	0 ^L−FL−FR^	5.7 ^L−FR^	0 ^L^	0 ^L^
	**Apiaceae**						
Apiaceae	Carrot Family	Apiáceas	Undetermined	3.7 ^L−FL−FR^	26.4 ^L−FL−FR^	4.7 ^L^	13.3 ^L^
*Pastinaca sativa*	Parsnip	Apio de campo	Introduced	0 ^L−FL^	1.9 ^L−FR^	0	0 ^L^
*Petroselinum crispum*	Garden Parsley	Perejil	Introduced	0 ^L−FL−FR^	0 ^L−FL−FR^	2.3 ^L^	0 ^L^
Asterales							
	**Asteraceae**						
*Ambrosia tenuifolia*	Lacy Ambrosia	Ambrosía de hojas finas	Native	0 ^L−FL−FR^	1.9 ^L−FL−FR^	0	0 ^L^
*Artemisia* sp.	Mugworts	Ajenjos	Introduced	0 ^L−FL−FR^	0 ^L−FL−FR^	2.3 ^FR^	0 ^L^
*Asteraceae*	Daisy Family	Asteráceas, compuestas	Undetermined	70.4 ^L−FL−FR^	90.6 ^L−FL−FR^	25.6 ^L^	73.3 ^L^
*Carthamus* sp.	Distaff Thistle	Cártamo	Introduced	63 ^L−FL−FR^	50.9 ^FR^	9.3 ^L^	53.3 ^L^
*Carthamus glaucus*	Mediterranean Thistle	Cardo alazor	Introduced	48.1 ^L−FL−FR^	43.4 ^FR^	9.3 ^L^	36.7 ^L^
*Carthamus lanatus*	Downy Safflower	Azotacristos	Introduced	66.7 ^L−FL^	64.2 ^FL−FR^	9.3 ^L^	60 ^L^
*Carthamus leucocaulos*	White-stem Distaff Thistle	Cardo	Presumed introduced	53.7 ^L−FL^	56.6 ^FL−FR^	7 ^L^	40 ^L^
*Centaurea* sp.	Knapweed	Abrepuños	Introduced	1.9 ^L−FL−FR^	5.7 ^FL−FR^	2.3 ^L^	10 ^L^
*Centaurea calcitrapa*	Red Star-thistle	Abrepuño, abrojo	Introduced	0 ^L−FL^	0 ^FL−FR^	0 ^L^	3.3 ^L^
*Cichorium intybus*	Belgium Endive	Achicoria, radicheta	Introduced	0 ^L^	1.9 ^L−FL−FR^	0	0 ^L^
*Conyza* sp.	fleabane	Hierba hedionda	Native	0 ^L−FL−FR^	1.9 ^L−FL−FR^	0 ^L^	0 ^L^
*Conyza bonariensis*	hairy fleabane	Rama negra	Native	0 ^L−FL−FR^	1.9 ^L−FL−FR^	0 ^L^	0 ^L^
*Cotula australis*	Annual Buttonweed	Motita, botón dorado, cotula, uña de gato	Introduced	0 ^L−FL−FR^	1.9 ^FL−FR^	0 ^L^	0 ^L^
*Cynara* sp.	Cynara	Cardos	Introduced	0 ^L−FL−FR^	13.2 ^FL−FR^	2.3 ^L^	0 ^L^
*Dahlia* sp.	Dahlia	Dalias	Ornamental	0 ^L−FL^	1.9 ^L−FL−FR^	0	0 ^L^
*Erigeron* sp.	Daisy	Margaritas	Native	0 ^L^	1.9 ^FL−FR^	0 ^L^	0 ^L^
*Facelis retusa*	Annual Trampweed	Facelis	Native	0 ^L−FL−FR^	0 ^FL−FR^	0 ^L^	10 ^L^
*Gazania* sp.	Gazania	Gazania	Introduced	0 ^L−FL−FR^	5.7 ^L−FL−FR^	0 ^L^	0 ^L^
*Helianthus annuus*	Annual Sunflower	Girasol	Cultivated	0 ^L−FL^	9.4 ^FL−FR^	0 ^L^	3.3 ^L^
*Hypochaeris* sp.	Cat’s Ear	Radichetas salvajes	Native	1.9 ^L−FL−FR^	5.7 ^L−FL−FR^	0	0 ^L^
*Lactuca sativa*	Garden Lettuce	Lechuga	Cultivated	9.3 ^L−FL−FR^	7.5 ^L−FL−FR^	7 ^L^	10 ^L^
*Solidago canadensis*	Canada Goldenrod	Vara de oro de Canadá, plumero Amarillo	Presumed introduced	0 ^L^	1.9 ^L−FL−FR^	0	0 ^L^
*Sonchus* sp.	Sow Thistle	Cerrajas	Introduced	0 ^L−FL−FR^	3.8 ^L−FL−FR^	0 ^L^	0 ^L^
*Sonchus asper*	Native Sow-Thistle	Cerraja, cerraja brava, cerraja espinosa, cerraja, yerba del pajarito, nilhué	Introduced	0 ^L−FL^	5.7 ^L−FL−FR^	0 ^L^	0 ^L^
*Taraxacum* sp.	Dandelions	Dientes de león, panaderos	Introduced	0 ^L−FL^	7.5 ^L−FL−FR^	0 ^L^	3.3 ^L−FL^
*Xanthium* sp.	Cocklebur	Abrojos	Undetermined	3.7 ^L^	11.3 ^L−FL−FR^	0 ^L^	6.7 ^L^

**Table 3:** Jaccard indices corresponding to the number of plant MOTUs found during the different life cycle stages of the Burrowing Parrot *Cyanoliseus patagonus* from El Cóndor, north-eastern Patagonia, Argentina

	Post-breeding	Winter	Pre-breeding
Breeding	26.5	30.2	30.6
Post-breeding	-	24.6	25.2
Winter	-	-	31

### Gap analysis

The accuracy of DNA metabarcoding and its taxonomic assignments depends on the completeness of the DNA barcode reference libraries such as NCBI GenBank for the organisms involved. However, these libraries often lack coverage of certain taxonomic groups (Leite et al. 2020; Specchia et al. 2020). To account for the possibility of such a gap in our study, in particular for native plants, we examined the extent to which plants found in the studied region are represented in the NCBI GenBank library. We used a list of native plants found in a region around El Cóndor, which is bordered by the localities of San Antonio Oeste (Río Negro) to the west, General Conesa (Río Negro) to the northwest, and Bahía San Blas (Buenos Aires) to the northeast (Table S3; Cintia V. Leder, unpublished). This list was compiled as part of previous studies conducted between 2009 and 2024. During visits to the sampling sites, preliminary surveys were conducted to verify the spatial consistency of the vegetation units, i.e., their overall uniformity in terms of physiognomy. Following the methodology proposed by Matteucci & Colma (1982) for estimating the number of sample units, five 100 m^2^ plots were selected. A floristic inventory was carried out in each plot, recording tree, shrub/subshrub, herbaceous, climbing, epiphyte, grass and cactus species. The taxonomic assignments in the list follow those in Anton and Zuloaga (2025), the Global Biodiversity Information Facility (2025), and Plants of the World Online (2025). To perform the gap analysis, we searched the NCBI GenBank library (https://www.ncbi.nlm.nih.gov/genbank/; accessed 22 October 2024) for reference sequences of each plant on the list using the following keywords: ‘internal transcribed spacer 2’ and ‘ITS2’. We recorded the presence of a reference sequence for each species and genus on the list.

### Plant phenology

In Table 2, we also provide details, where available, of the phenology phases of plants in north-eastern Patagonia that correspond to the stages of the parrot life cycle. For instance, we show that *Amelichloa caudata*, a type of ricegrass, underwent through the leaf stage, the flowering development and fruit growth and seed release during the Burrowing Parrot breeding season. Fruit growth and seed release continued during the post-breeding season of the parrots, while the plant remained in the leaf stage throughout winter and the pre-breeding stages of the parrot life cycle. To gather the phenological data for the species or taxonomic groups identified from the MOTUs, an extensive search was conducted in specific literature from central and southern Argentina (Cano 1988; Forcone 2003, Prina et al. 2015; Sanhueza et al. 2016) and in online public databases such as the Sistema de Información de Biodiversidad (2025) and the Sistema Nacional de Vigilancia y Monitoreo de Plagas (2025). The phenological stages were then adjusted for the region based on our own observations (Cintia V. Leder and team) over more than 15 years of fieldwork.

## Results

### Diet composition

We identified 202 valid plant MOTUs belonging to the class Spermatopsida in the Burrowing Parrot’s diet. In Table 2, we provide taxonomic details for each MOTU, categorized them according their likely source, and the corresponding frequency of occurrence for the different life cycle stages. The MOTUs belonged to 23 orders and 48 families (Table 2). We identified 99 MOTUs at the species level, 73 at the genus level, and 30 at the family level.

Burrowing parrots consumed 77 plant taxa during breeding, 172 during post-breeding stage, 72 in winter, and 70 during pre-breeding (Table 2). They consumed more different plants during the post-breeding stage than during the other life cycle stages (Kruskal-Wallis rank sum test, χ^2^ = 80.7, df = 3, *p* < 0.001; Fig. 1). In Table 3, we present the Jaccard indices corresponding to the number of plant MOTUs found during the Burrowing Parrot’s life cycle stages. The similarity coefficients ranged from 24.6% to 31.0%, with the lowest value found between post-breeding and winter, and the highest between winter and pre-breeding (Table 3).

Of the 202 valid MOTUs, 80 (40%) corresponded to introduced plants, 9 (4.5%) to presumed introduced, 24 to ornamental (12%), 14 to cultivated (6.9%), 26 to native plants (13%), and the rest (49; 24%) could not be labeled with certainty. When we considered individual samples, the observed frequency of distribution of native, introduced, presumed introduced, cultivated, and ornamental plants varied significantly across the different life cycle stages of the Burrowing Parrot (Table 4; Pearson’s χ^2^ = 35.8, df = 3, *p* < 0.001). Most of the samples included introduced plants in all life cycle stages such as *Cynodon* sp., *Tribulus terrestris*, *Diplotaxis* sp., *Brassica rapa* and *Carthamus* spp. (Tables 2 and 3). Cultivated plants including *Avena sativa*, *Hordeum vulgare*, and *Triticum* sp. were present in most samples during breeding, post-breeding and winter, while native plants like *Festuca* sp., *Condalia* sp., Rhamnaceae and *Hypochaeris* sp. were present in the majority of samples during the post-breeding stage (Tables 2 and 4).

**Table 4:** Number of samples containing plant MOTUs split according to their likely source during the different life cycle stages of the Burrowing Parrot *Cyanoliseus patagonus* from El Cóndor, north-eastern Patagonia, Argentina. *n* = number of fecal samples from which DNA was extracted

	*n*	Native	Cultivated	Introduced	Ornamental
Breeding	53	10	42	49	13
Post-breeding	53	35	51	53	30
Winter	42	10	35	40	12
Pre-breeding	30	5	17	29	7

**Fig. 5:**
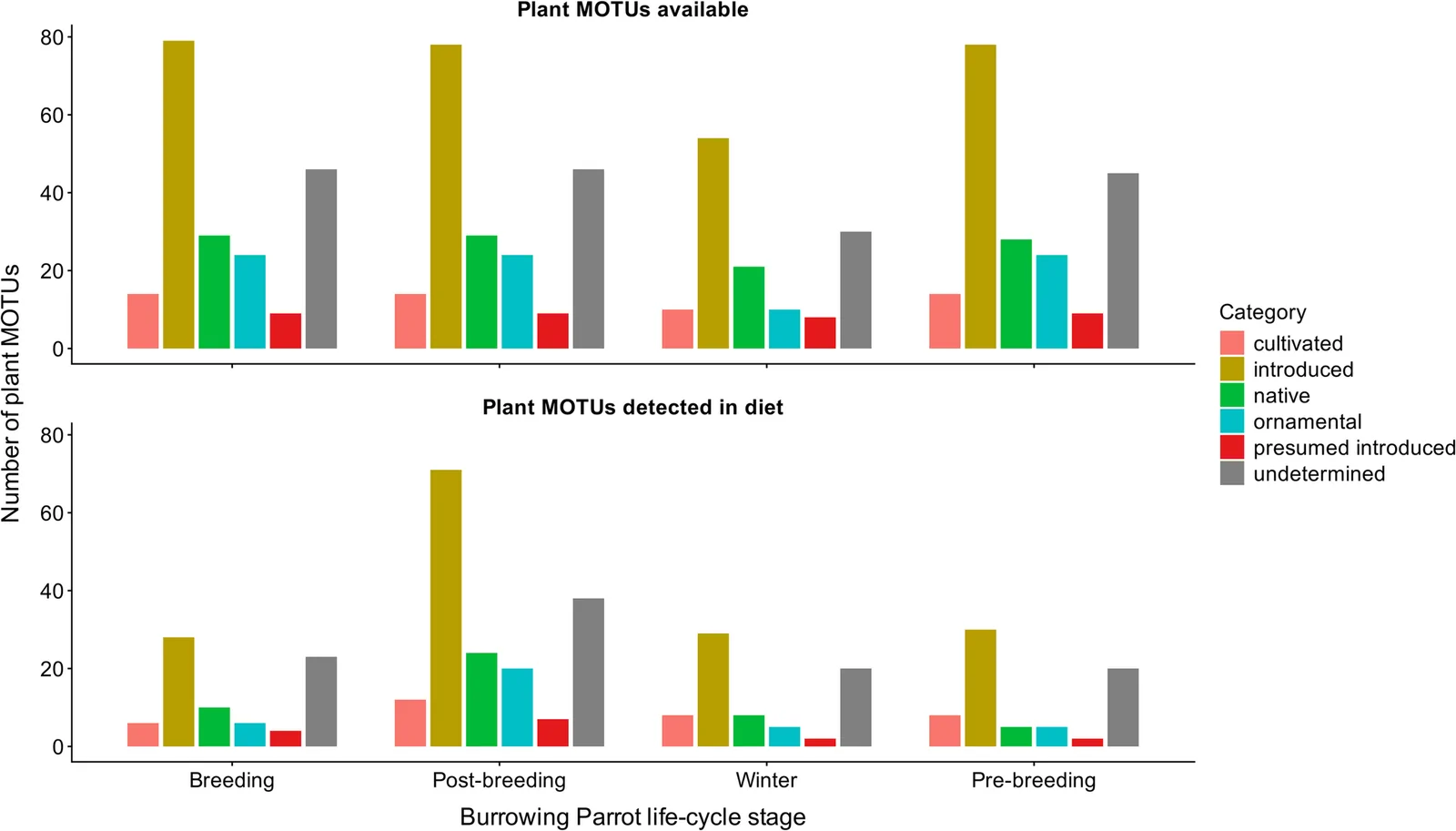
Temporal correspondence between plant (class Spermatopsida) molecular operational taxonomic units (MOTUs) in the diet composition and plant phenology across the life-cycle stages of Burrowing Parrots from El Cóndor in north-eastern Patagonia, Argentina. Bars show the frequency of occurrence of MOTUs detected in fecal samples for each life-cycle stage, while colors indicate plant category (native, introduced, cultivated, undetermined, and presumed introduced). For further details see also Table 2

Both at the species (Fig. 2) and genus level (Fig. 3), we observed considerable overlap in the Spermatopsida valid MOTUs identified in the Burrowing Parrot’s diet during breeding, post-breeding, and pre-breeding, but far less overlap between winter and the other life cycle stages (PERMANOVA, at the species level: F_162,3_ = 6.9, *P* < 0.001, at the genus level: F_158,3_ = 6.6, *P* < 0.001). Table 2 and Figs. S1 and S2 revealed that mostly introduced and ornamental Spermatopsida such as *Picea glauca* (Pinaceae), *Eriocoma* sp., *Holcus lanatus*, *Lolium perenne*, *Schismus* sp. (Poaceae), *Atriplex semibaccata* (Amaranthaceae), *Carpinus betulus* (Betulaceae), *Brassica oleracea* (Brassicaceae), *Artemisia* sp. (Asteraceae) were present in winter samples, but not in some of the other life cycle stages. Among the species with the highest frequency of occurrence, and present across all parrot’s life cycle stages, we observed mostly introduced species: *Pinus sylvestris* (Pinaceae; ornamental), *Avena sativa*, *Hordeum vulgare*, *Triticum aestivum* (Poaceae; cultivated), *Salsola tragus* (Amaranthaceae; introduced), *Tribulus terrestris* (Zygophyllaceae; introduced), *Betula pendula* (Betulaceae; introduced), *Brassica rapa* (Brassicaceae; introduced), *Carthamus glaucus*, *Carthamus lanatus*, *Carthamus leucocaulos* (Asteraceae; introduced) (Table 2). At the family level (Fig. 4), we also observed considerable overlap in the Spermatopsida valid MOTUs during breeding, winter, and pre-breeding, but far less overlap between post-breeding and the other main life cycle stages (PERMANOVA: F_159,3_ = 6.7, *P* < 0.001). MOTUs corresponding to the families Polygonaceae, Aizoaceae, Ulmaceae, Juglandaceae, and Pittosporaceae were exclusively present in samples from the post-breeding life cycle stage (Table 2, Fig. S3). Urticaceae were present only in winter samples (Table 2). In Fig. 5, we show the temporal correspondence between plants identified in the parrot’s diet and plant phenology (see also Table 2) across the life-cycle stages of Burrowing Parrots from El Cóndor.

### Gap analysis

The list of native plants found in a region around El Cóndor included 126 plants identified at the species level, and 8 at the genus level (Table S3). At the species level, we found that for 69% of the plants at least one reference sequence was available in GenBank library at the time of our search. At the genus level, at least one reference sequence was available for 97% of the plants in our list. The taxa with the larger gaps in the reference libraries belonged to the families Asteraceae (12), Cactaceae (4), Bromeliaceae (3) and Fabaceae (3) (Table S3).

### Bilateralia present

Using a primer targeting Bilateralia, we identified 31 valid MOTUs. In Table 5, we provide taxonomic details for each MOTU, ecological categories, and the corresponding frequency of occurrence. The Bilateralia MOTUs belong to nine classes, 18 orders, and 25 families (Table 4). Of these, we were able to identify 24 MOTUs at the family level, seven at the order level and one at the class level. From the 31 valid MOTUs, three could be labeled as intestinal parasites (Hymenolepididae, Onchobothriidae, and Capillariidae), one as an oral cavity parasite (Gongylonematidae), one as an ingested ectoparasite (Cimicidae), four as bycatch (Anguinidae, Naididae, Cecidomyiidae, Aphididae), 21 as presumed bycatch, and one as presumed ingested ectoparasite (Table 4).

**Table 5:** Valid molecular operational taxonomic units (MOTUs) of Bilateralia present in the diet of the Burrowing Parrot *Cyanoliseus patagonus* from El Cóndor, north-eastern Patagonia, Argentina, their lowest taxonomic assignment and their frequency of occurrence (% of samples)

Class	Order	Family	MOTUname	Category	Frequency of occurrence (%)
Arachnida	Araneae	Deinopidae	Deinopidae	Presumed bycatch	1.1
Arachnida	Ixodida	Ixodidae	Ixodidae	Presumed bycatch	0.6
Arachnida	Pseudoscorpiones	-	Pseudoscorpiones	Presumed bycatch	0.6
Arachnida	Sarcoptiformes	Hemileiidae	Hemileiidae	Presumed bycatch	0.6
Arachnida	Sarcoptiformes	-	Sarcoptiformes	Presumed bycatch	0.6
Arachnida	Trombidiformes	Demodecidae	Demodecidae	Presumed ingested ectoparasite	0.6
Arachnida	Trombidiformes	Raphignathidae	Raphignathidae	Presumed bycatch	0.6
Arachnida	Trombidiformes	Tetranychidae	Tetranychidae	Presumed bycatch	1.7
Branchiopoda	Diplostraca	Daphniidae	Daphniidae	Presumed bycatch	1.7
Branchiopoda	-	-	Branchiopoda	Presumed bycatch	1.1
Cestoda	Cyclophyllidea	Hymenolepididae	Hymenolepididae	Intestinal parasite	0.6
Cestoda	Onchoproteocephalidea	Onchobothriidae	Onchobothriidae	Intestinal parasite	0.6
Chilopoda	Geophilomorpha	-	Geophilomorpha	Presumed bycatch	0.6
Chromadorea	Rhabditida	Anguinidae	Anguinidae	Bycatch	2.8
Chromadorea	Rhabditida	Aphelenchidae	Aphelenchidae	Presumed bycatch	2.8
Chromadorea	Rhabditida	Gongylonematidae	Gongylonematidae	Oral cavity parasite	0.6
Chromadorea	Rhabditida	Rhabditidae	Rhabditidae	Presumed bycatch	1.1
Chromadorea	Rhabditida	Tylenchidae	Tylenchidae	Presumed bycatch	0.6
Clitellata	Tubificida	Naididae	Naididae	Bycatch	0.6
Collembola	Symphypleona	-	Symphypleona	Presumed bycatch	0.6
Enoplea	Trichinellida	Capillariidae	Capillariidae	Intestinal parasite	1.7
Insecta	Diptera	Cecidomyiidae	Cecidomyiidae	Bycatch	2.2
Insecta	Diptera	-	Diptera	Presumed bycatch	0.6
Insecta	Diptera	Sarcophagidae	Sarcophagidae	Presumed bycatch	0.6
Insecta	Hemiptera	Aphididae	Aphididae	Bycatch	0.6
Insecta	Hemiptera	Cicadellidae	Cicadellidae	Presumed bycatch	0.6
Insecta	Hemiptera	Cimicidae	Cimicidae	Ingested ectoparasite	0.6
Insecta	Hemiptera	Lygaeidae	Lygaeidae	Presumed bycatch	1.1
Insecta	Lepidoptera	-	Lepidoptera	Presumed bycatch	3.3
Insecta	Lepidoptera	Pieridae	Pieridae	Presumed bycatch	1.1
Insecta	Thysanoptera	Thripidae	Thripidae	Presumed bycatch	1.1

## Discussion

Using NGS of DNA in fecal samples we identified a large variety of plant MOTUs (Table 2) in the diet composition of Burrowing Parrots over the annual cycle. They consumed the greatest diversity of MOTUs during the post-breeding stage (Figs. 1 and 5), a period when most of the plants recorded in our study grow fruits and release seeds (Table 2). This is also a time of the year critical for the survival of Burrowing Parrot juveniles, which strongly depend on adequate food availability after fledging to be able to prepare for the harsh Patagonian winter weather (Masello and Quillfeldt 2004; Naef-Daenzer et al. 2016, Tang et al. 2023). We observed considerable overlap in the valid MOTUs identified during the pre-breeding, breeding and post-breeding stages, but less overlap with the winter stage (Figs. 2 and 3). This may indicate that the parrots feed on different parts of the same plant (leaves, flowers, fruits) as the seasons progress, while many of these plants (or their edible parts) are not readily available to the parrots in winter (Table 2). These are frequent patterns that have also been observed in other birds, including Psittaciformes (Powlesland et al. 1997; Oliveira et al. 2002; Díaz et al. 2012; Cabodevilla et al. 2021; Ragusa-Netto 2022; Tang et al. 2023). However, it is important to note that the methods used in this study do not enable us to determine the origin of the DNA within the plant. Direct observations of foraging Burrowing Parrots in the field are needed to properly investigate this.

Notably, we found that 40% of plant MOTUs consumed by Burrowing Parrots over the year were introduced plants, with a further 4.5% corresponding to presumed introduced (Table 2). Moreover, most of the samples included introduced plants at all Burrowing Parrot life cycle stages (Table 4; Fig. 5). By contrast, only 13% of the MOTUs corresponded to native plants (Table 2), and fewer samples included them (Table 4; Fig. 5). This is unexpected, given that previous information on the diet of Burrowing Parrots suggested that berries, fruits and seeds of native trees and grasses in the Monte vegetation played a key role in their diet (Hudson 1923; Wetmore 1926; Bucher et al. 1987; de la Vega 2003; Masello et al. 2006a; de la Peña 2011; Sánchez et al. 2016). There may be various reasons for the difference observed between previous and current study.

The large proportion of introduced plants in the diet of Burrowing Parrot could be the result of a potential methodological bias. We collected the samples in the evening, when large parrot flocks roost on the power lines inside the village of El Cóndor (Masello et al. 2006a). Consequently, our results could have been biased towards items consumed in the latter part of the day, as the parrots make their way to the roosting place in the village (Masello et al. 2006a, 2025). The landscape surrounding the village is the most intensively modified in the region, and the occurrence of introduced and ornamental plants appears to be highest close to the village (author’s pers. observ.). However, previous observations of the behavior of Burrowing Parrots offer no support to this potential bias. Investigating the timing of movements, we conducted observations of Burrowing Parrots flying from the foraging locations back to the colony and the roosting place in El Cóndor during five breeding seasons (Masello et al. 2025). During these movements, the parrots consistently flew over fields a few hundred meters north of El Cóndor village, without stopping to feed in any of them. Moreover, we have previously shown that the parrots forage in locations situated between 58 km northwest and 66 km northeast of the colony (Masello et al. 2006a; Sánchez et al. 2016). In addition, previous studies investigating the transit time of food items in Psittaciformes also counteract a potential bias towards items taken by the parrots late in the day. They showed that the entire digestive tract of corn-fed Budgerigar *Melopsittacus undulatus* empties within 26 h (Sakurai et al. 1998; Koutsos et al. 2001). Consequently it is most likely that the MOTUs we detected were consumed by the Burrowing Parrots during the previous entire day of foraging.

The proportion of introduced plants in the Burrowing Parrot’s diet may have been inflated due to a gap in DNA barcode reference libraries in the NCBI GenBank for native plants in northeastern Patagonia. To account for such possibility, we investigated how well the plants found in the studied region are represented in the NCBI GenBank library. Our gap analysis revealed that 69% of Spermatopsida in northeastern Patagonia are represented in GenBank at species level and 97% at genus level. As 85% of our MOTUs could be identified at genus level or above, the completeness of the reference libraries in GenBank is likely to be adequate. Compared with the much larger gaps found for other organisms and locations (Leite et al. 2020; Specchia et al. 2020), the small gap detected in our study at the genus level is unlikely to fully explain the high proportion of introduced plants observed in the Burrowing Parrot’s diet.

Environmental degradation may provide a more plausible explanation. The land neighboring the study area has been classified as part of the southern ‘Monte’ (Bisigato et al. 2009), the ‘Espinal’ (Abraham et al. 2009) or a transition zone between the two phytogeographical provinces (Torres Robles & Rodríguez 2023). Historically, the area was a scrubland characterized by different types of desert shrubs and grasses (Kröpfl et al. 2005, 2015, Roig et al. 2009). But, since the 1970s, a combination of increased precipitation at the beginning of that period and the severe clearance of natural vegetation have enabled agricultural intensification (Gabella 2014; Sánchez et al. 2016). Particularly, to the northeast of El Cóndor village, in the Patagones department (Buenos Aires province), the natural vegetation that covered 65% of the land in 1975 has been reduced to only 20 to 29%, according to estimations (Pezzola et al. 2004; Gabella 2014; Cosentino et al. 2021; Torres Robles & Rodríguez 2023). Gaspari et al. (2021) reported that in only three years (2015–2018), 216,961 hectares of natural vegetation were cleared in Patagones. In the department of Adolfo Alsina (Río Negro province), to which El Cóndor village belongs, Lini (2008) reported that 48,864 hectares (of a total 881,300) were cleared between 1986 and 2006. Meanwhile, Rodríguez (2017) demonstrated that 483,900 hectares show strong signs of degradation. Unfortunately, regardless of the devastating implications for conservation, economy, and public health, already identified over 20 years ago (Pezzola et al. 2004; Masello et al. 2006a), the clearing of natural vegetation continues to this day (Masello et al. 2025). Consequently, the land surrounding the village and the El Cóndor Burrowing Parrot colony is now primarily a mixture of cropland and low-density cattle grazing areas (Gabella 2014; Sánchez et al. 2016). Landscape-scale disturbances, like those ongoing in Patagones and Adolfo Alsina, can disrupt the resident plant community and facilitate the spread of alien species (DeGasperis and Motzkin 2007; Mosher et al. 2009; Vilà and Ibáñez 2011; Mattingly and Orrock 2013). Furthermore, the success of alien species is often associated with the resulting reduction in local plant species richness and productivity (Mattingly and Orrock 2013). Under these circumstances, Burrowing Parrots appear to be switching from a predominantly Monte/Espinal-based diet in the past (Hudson 1923; Wetmore 1926; Bucher et al. 1987; Masello et al. 2006a; de la Peña 2011; de la Vega 2003; Sánchez et al. 2016), to one including a large proportion of introduced species (Tables 2 and 4; Fig. 5). Further research is needed to determine whether this behavior is an adaptation of the Burrowing Parrot and a sign of behavioral flexibility or whether it is a suboptimal strategy with negative consequences for the persistence of the colony at El Cóndor. Either way, our results contribute to the growing body of evidence indicating the increasing degradation of the natural habitats of northeastern Patagonia (Pezzola et al. 2004; Lini 2008; Boeri et al. 2014; Gabella 2014; Cosentino et al. 2021; Gaspari et al. 2021; Torres Robles & Rodríguez 2023). Furthermore, by studying the diet of Burrowing Parrots, we have documented the likely introduction of nine plant species not previously recorded in the region (Table 2). This should serve as a warning about the urgent need to protect the remaining Monte in the region, and to monitor introduced plants in case they become invasive if environmental conditions deteriorate further (Vilà and Ibáñez 2011; Mattingly and Orrock 2013).

The low proportion of native plants may also have been related to the persistent La Niña event that affected north-eastern Patagonia prior to and during the study period (Li et al. 2022; Jiang et al. 2023). The region experiences severe droughts during the La Niña phase of the El Niño–Southern Oscillation (ENSO), reducing terrestrial gross primary production and causing food shortages for wildlife (Golluscio et al. 1998, Holmgren et al. 2001, Masello and Quillfeldt 2004; Masello et al. 2008; Zhang et al. 2019). Burrowing Parrots respond to such adverse conditions by delaying breeding and engaging in brood reduction, resulting in fewer nestlings per nest fledging (Masello and Quillfeldt 2002, 2004). Moreover, the La Niña conditions had a negative impact on nestling growth (Masello and Quillfeldt 2003, 2004), and reduced the proportion of active nests at the El Cóndor colony (Masello and Quillfeldt 2002; Masello et al. 2025). Similarly, in the breeding season previous to this study (2020/2021), a high mortality among adult Burrowing Parrots was observed at El Cóndor, likely due to a food shortage triggered by La Niña (Masello et al. 2025). Although these observations suggest a potential connection between our results and lower availability of native plants under La Niña, a causal relationship cannot be inferred from a single-year sampling period. A multi-year study would be required to properly investigate this link. In fact, the expected increase in the frequency of La Niña events (Cai et al. 2015; Marjani et al. 2019; Geng et al. 2023) makes such a study essential in order to inform any future conservation measures.

We found that 6.9% of the MOTUs consumed by Burrowing Parrots corresponded to cultivated plants (Table 2; Fig. 5). Burrowing Parrots have been considered an agricultural pest, and persecuted during decades (Bucher and Bedano 1976; Bucher 1984; Masello et al. 2006a, Failla et al. 2008). However, systematic evaluations have revealed that damage to field crops is negligible and of little economic significance (Sánchez et al. 2016). Consequently, the species has been removed from all former lists of avian pests subject to control (e.g. Ministerio de Ambiente y Desarrollo Sustentable and Aves Argentinas 2017). Still, Burrowing Parrots have been found to consume cultivated grain that has been spilled during harvesting, as well as unharvested grain from cultivated pastures (Sánchez et al. 2016). In line with these previous findings, we detected that Burrowing Parrots consumed MOTUs that are common cultivated pastures in Argentina (e.g. *Avena* spp., *Beta vulgaris*, *Holcus* sp., *Hordeum vulgare*, *Sorghum bicolor*, *Triticum aestivum*; Table 2; Brizuela and Cangiano 2011) across all stages of the parrot’s life cycle (Table 4; Fig. 5). Data from the Instituto Nacional de Estadísticas y Censos (2025) and the Ministerio de Agricultura, Ganadería y Pesca (2025) indirectly support our interpretation that the MOTUs detected mostly correspond to cultivated pastures. According to these official sources, 72% (22,298 hectares) of the cultivated area in the Adolfo Alsina department was used for cultivated pastures, primarily *Avena* spp. and *Hordeum vulgare*. In the Patagones department, 54% of cultivated land (480,823 hectares) was used for pasture, mainly consisting of *Avena* spp. and *Hordeum vulgare*. As these two departments, mainly Adolfo Alsina, surround the El Cóndor colony it is likely that the MOTUs we classified as ‘cultivated’ correspond mostly to cultivated pastures. However, as described in Sánchez et al. (2016), the Burrowing Parrots may also have taken grain spilled during harvesting and grain from the borders of crop fields.

A previous study that searched for intestinal parasite eggs and oocysts in fecal samples from Burrowing Parrots found none (Masello et al. 2006b). These negative results may have been caused by the use of a suboptimal flotation solution or by sampling high-quality individuals (Masello et al. 2006b). However, the use of NGS of DNA in fecal samples allowed us to identify some parasites present in the digestive tract of Burrowing Parrots. One of them, belonging to the family Gongylonematidae (Table 5), infects the oral cavity of birds, and can lead to starvation and death (Esperón et al. 2013). During the breeding season 2020–2021, a large mortality of Burrowing Parrots affected the El Cóndor colony (Masello et al. 2025). At least 1,050 dead adult parrots were found, compared with the 20–30 that typically are found each year. While starvation related to weather conditions could have been the cause of the deaths, veterinary experts suggested that the primary cause was an infection (Masello et al. 2025). Here, we can only speculate about the possibility of a widespread Gongylonematidae infection causing the mortality event. However, based on our current findings, this is a possibility that must be investigated in the future. Additionally, we detected three intestinal parasites belonging to the Hymenolepididae and Onchobothriidae (Cestoda), and Capillariidae (Enoplea). Hymenolepididae tapeworms are common in both wild and domestic waterfowl (Guo 2016; Drago et al. 2021; Eslahi et al. 2024). These parasites have previously been recorded in Argentinian birds, including some widely distributed in the Río Negro province (Tinamiformes, Charadriiformes), where the El Cóndor Burrowing Parrot colony is located. However, they have not been found in Psittaciformes previously (Drago et al. 2021). The primary hosts of the Onchobothriidae are freshwater fish, but they have also been reported in amphibians, lizards, snakes and marsupials (Caira et al. 2017; de Chambrier et al. 2017). Until our study, Onchobothriidae had not been reported in Psittaciformes or any other bird. However, it is expected that the number of bird hosts will increase as more extensive research is conducted (Carlson et al. 2020). It is worth mentioning that there is little evidence to suggest that adult Cestoda have an adverse effect on birds unless present in massive numbers (McLaughlin 2008). Capillaridae are thin nematodes previously found in captive Psittaciformes, most probably transmitted through the contact with captive Columbiformes (Kajerová and Baruš 2005; Yabsley 2008). A similar scenario could be possible for the Burrowing Parrots of El Cóndor. At the end of the nesting season, strong winds and attacks by Kelp Gulls *Larus dominicanus* and Peregrine Falcons *Falco peregrinus* prevent some Burrowing Parrot fledglings from performing the demanding first flight from their nest at the cliff to the top of the cliff, where their parents usually await them. Neighbors from El Cóndor village regularly find these fledglings, take them home, where other domestic birds are common, and care for them temporarily. Finally, they release the surviving Burrowing Parrot fledglings, which potentially could carry with them infections. Despite extensive campaigns to stop this practice, it still persists. Fortunately, although the prevalence of Capillaridae may be high in some cases, clinical disease in wild birds is rare, affecting individuals more than populations (Yabsley 2008).

## Conclusions

Next-generation sequencing of DNA in fecal samples allowed us to identify the dietary composition of the Burrowing Parrots from El Cóndor over the annual cycle, a valuable information with ramifications for future research. The results were unexpected, suggesting a switch from a diet predominantly based on plants from the Monte/Espinal in the past to a current diet primarily composed of introduced plants. This change could be a demonstration of the Burrowing Parrots’ behavioral flexibility in adapting to the severe environmental degradation in northeastern Patagonia. Or a sign of a suboptimal strategy that could negatively impact the persistence of the El Cóndor colony. As the clearance of the Monte vegetation continues to date, and the El Cóndor colony comprises the majority of the Burrowing Parrot breeding population (71% of the global breeding pairs; Masello et al. 2025), a close monitoring of both population sizes and diet is required to inform necessary conservation measures. Similarly relevant is our finding of a Gongylonematidae parasite. Future investigation into its possible connection with the Burrowing Parrot mortality of 2020–2021 could help to prevent a similar event in the future.

## Supplementary Information

Below is the link to the electronic supplementary material.

Supplementary Material 1

Supplementary File 2 Species level identity of the plant items (class Spermatopsida) consumed by the Burrowing Parrot *Cyanoliseus patagonus* from El Cóndor in north-eastern Patagonia, Argentina, collapsed in two dimensions (MDS1 and MDS2), according to the main life cycle stages (breeding, post-breeding, winter, pre-breeding), using non-metric multidimensional scaling of molecular operational taxonomic units

High Resolution Image

Supplementary File 3 Genus level identity of the plant items (class Spermatopsida) consumed by the Burrowing Parrot *Cyanoliseus patagonus* from El Cóndor in north-eastern Patagonia, Argentina, collapsed in two dimensions (MDS1 and MDS2), according to the main life cycle stages (breeding, post-breeding, winter, pre-breeding), using non-metric multidimensional scaling of molecular operational taxonomic units

High Resolution Image

Supplementary File 4 Family level identity of the plant items (class Spermatopsida) consumed by the Burrowing Parrot *Cyanoliseus patagonus* from El Cóndor in north-eastern Patagonia, Argentina, collapsed in two dimensions (MDS1 and MDS2), according to the main life cycle stages (breeding, post-breeding, winter, pre-breeding), using non-metric multidimensional scaling of molecular operational taxonomic units

High Resolution Image

## Data Availability

Sequences will be uploaded to the NCBI Sequence Read Archive upon manuscript acceptance. Additional data supporting the conclusions of this article are available or cited within the article and its Supplementary Materials.
